# Motivation for Rehabilitation in Patients With Subacute Stroke: A Qualitative Study

**DOI:** 10.3389/fresc.2021.664758

**Published:** 2021-06-07

**Authors:** Taiki Yoshida, Yohei Otaka, Rieko Osu, Masashi Kumagai, Shin Kitamura, Jun Yaeda

**Affiliations:** ^1^Tokyo Bay Rehabilitation Hospital, Narashino, Japan; ^2^Graduate School of Human Sciences, Waseda University, Tokorozawa, Japan; ^3^Faculty of Rehabilitation, School of Health Sciences, Fujita Health University, Toyoake, Japan; ^4^Department of Rehabilitation Medicine I, Fujita Health University School of Medicine, Toyoake, Japan; ^5^Faculty of Human Sciences, Waseda University, Tokorozawa, Japan; ^6^Graduate School of Comprehensive Human Science, University of Tsukuba, Tokyo, Japan

**Keywords:** motivation, stroke, rehabilitation, qualitative study, age

## Abstract

**Background:** Motivation is essential for patients with subacute stroke undergoing intensive rehabilitation. Although it is known that motivation induces behavioral changes toward rehabilitation, detailed description has been lacking. Motivation can be intrinsic or extrinsic; however, it is unclear which type of factors mainly motivates patients' daily rehabilitation.

**Purpose:** This study aimed to examine the factors influencing patients' motivation and to explore the behavioral changes induced by motivation, especially age-related differences.

**Method:** Twenty participants (mean age 65.8 years [standard deviation 13.7]) who had a subacute stroke and underwent rehabilitation at a convalescent hospital were recruited using convenience sampling. Semi-structured interviews were conducted by an occupational therapist with an interview topic guide regarding factors influencing motivation and how it affects behavioral change. Interviews were recorded, transcribed to text, and analyzed by three occupational therapists using thematic analysis. The participants were divided into two groups: aged patients (aged ≥ 65 years) and middle-aged patients (aged < 65 years), and data were analyzed according to the groups. This study was conducted according to the consolidated criteria for reporting qualitative research.

**Results:** Seven core categories were identified as factors influencing patients' motivation: patients' goals, experiences of success and failure, physical condition and cognitive function, resilience, influence of rehabilitation professionals, relationships between patients, and patients' supporters. The first four and last three core categories were further classified as personal and social-relationship factors, respectively. The categories related to intrinsic motivation such as enjoyment of rehabilitation itself were not derived. In both age-groups, motivation affected the frequency of self-training and activity in daily lives. In some aged patients, however, high motivation restrained their self-training to conserve their physical strength for rehabilitation by professionals. Some aged patients do not express their high motivation through their facial expressions and conversations compared to middle-aged patients; therefore, motivation is not always observable in aged patients.

**Conclusions:** Interventions tailored to extrinsic factors are important for maintaining patients' motivation. Observational evaluation may lead to mislabeling of their motivation, especially for aged patients. Rehabilitation professionals should use validated evaluation scales or patients' narratives to assess patients' motivation.

## Introduction

Stroke patients undergoing rehabilitation are required to be active participants in their treatment, and motivation is an essential factor for active participation. Self-determination theory (SDT) is a commonly known motivational theory (1, 2) and is applied in rehabilitation fields (3, 4). Conventionally, motivation is divided into extrinsic motivation and intrinsic motivation. Extrinsic motivation involves doing something because it leads to a separable consequence; that is, the goal is separate from the activity itself. In contrast, intrinsic motivation involves doing something because it is interesting and enjoyable in itself (1, 2). According to this classification, rewards such as functional improvement and praise from medical staff and family members can be categorized as extrinsic motivation, and enjoyment of rehabilitation itself can be considered intrinsic motivation. In previous studies, motivation for rehabilitation was reported to be affected by extrinsic factors such as environmental factors (5–7), individual beliefs (6–10), and support from medical staff and family (7, 10–13). Since the majority of stroke patients in the convalescent phase attend rehabilitation to return to their before-stroke-onset lives, a form of extrinsic motivation, we do not expect that intrinsic motivation is a major part of their commitments. Especially subacute stroke patients who are in convalescent phase are considered to be more affected by extrinsic motivation than chronic patients, and since their physical functions are relatively more changeable than that of chronic stroke patients, it is easier for them to earn rewards, i.e., functional recovery. Thus, we hypothesize that subacute stroke patients undergo rehabilitation based mostly on extrinsic factors such as functional improvement.

Motivation can induce behavioral changes. In rehabilitation, motivation is suggested as a predictor of functional outcomes (14). The motivation for rehabilitation is reported to influence physical activity (8, 15) and participation in training (16, 17). Thus, motivation can be expressed as physical activity and attitude toward rehabilitation. Therefore, medical staff tend to label the patients' motivation based on the activity in their daily lives, their attitude, and compliance with rehabilitation (7). However, a previous qualitative study pointed out a mismatch between motivation and activity and warned that observational evaluation of patients' motivation by medical staff may sometimes lead to mislabeling (7). Stroke patients in convalescent rehabilitation hospitals are required to pursue intensive rehabilitation training. Since the physical strength and capacity of aged patients tend to be less than that of younger patients (18), they may not have enough capacity to actively engage in physical activities or express a positive attitude toward rehabilitation. If medical staff label patients' motivation according to daily activity or attitude, those patients are mislabeled as being unmotivated. To correctly evaluate these patients, a detailed description of behavioral change, especially, age-related differences in their behavior, is required.

The purpose of this study is as follows: first, we clarified the factors influencing patients' motivation for rehabilitation in the convalescent rehabilitation hospital. In particular, this study aimed to examine which type of factors, extrinsic or intrinsic, mainly motivates patients' daily rehabilitation. Second, we hypothesized that the behavioral changes influenced by motivation may differ in aged patients and middle-aged patients; thus, we analyzed the behavioral changes among different age-groups.

Although several evaluation scales of motivation for rehabilitation have been reported (19–22), no validated evaluation scale for rehabilitation explicitly contains items related to intrinsic motivation. Furthermore, questionnaires cannot provide detailed descriptions about the patients' behavioral changes in daily life. Thus, we adopted a qualitative method. Qualitative methods are appropriate for investigating topics that are poorly understood (23). Semi-structured interviews are based around open-ended questions that define the area to be explored; then, the interviewer and interviewee pursue an idea in more detail (23). In order to investigate the factors influencing motivation and patients' behavioral changes in detail, it is necessary to understand patients' narrative. Therefore, we employed semi-structured interviews to elucidate participants' motivation for rehabilitation. We also adopted thematic analysis, since it provides a more detailed and nuanced account of themes within the data (24, 25).

## Materials and Methods

This study was conducted according to the consolidated criteria for reporting qualitative research [COREQ; (26)]. The study protocol was approved by the Ethics Committee of Tokyo Bay Rehabilitation Hospital (No. 144) and the Ethics Committee of Waseda University (No. 2019-059). All participants provided written and verbal informed consent before participating in this study.

### Sample

Participants were recruited from those who were admitted to the Tokyo Bay Rehabilitation Hospital in Japan, which had three convalescent rehabilitation wards with 160 beds. The inclusion criteria were as follows: a Mini-Mental State Examination score of 24 or more and having had their first-ever hemiparetic stroke in the subacute phase (27). Patients who were diagnosed by their doctors as having aphasia, depression, or cognitive impairments, and therefore would have difficulty participating in the study, were excluded. Twenty-two Japanese patients who met the inclusion criteria were recruited through convenience sampling; of these, 20 agreed to participate (10 aged < 65 years). All participants were assessed with the Functional Independence Measure (FIM) (28) by trained nurses to evaluate their functional status. Sample characteristics are presented in Table 1.

**Table 1:** Sample characteristics.

	**Aged < 65 years (*n* = 10)**	**Aged ≥ 65 years (*n* = 10)**	***P*-value**
Gender, male/female, *n*	7/3	6/4	0.639
Age, mean (standard deviation)	53.5 (6.8)	78.0 (4.0)	<0.001^†^
Side of paresis (right/left/bilateral), *n*	5/5/0	4/5/1	0.574
Days from stroke onset to interview, mean (standard deviation)	84.7 (36.3)	74.5 (25.0)	0.597
Days from admission to interview, mean (standard deviation)	53.3 (31.0)	46.7 (24.4)	0.705
Total hospitalization days, mean (standard deviation)	86.9 (46.5)	86.9 (46.5)	0.472
Number of family members living together, median (interquartile range)	1 (0–2)	1 (1–1)	0.386
Functional Independence Measure total score, median (interquartile range)	117 (111–123)	109 (96–115)	0.088
Functional Independence Measure motor score, median (interquartile range)	83 (82–88)	80 (70–82)	0.103
Mini-Mental State Examination, median (interquartile range)	29 (29,30)	28 (28,29)	0.137
Living place before onset, home/not at home	10/0	10/0	
Working status before stroke, yes/no	9/1	3/7	0.006^†^

† *Significant difference between group aged < 65 years and aged ≥ 65 years*.

### Study Setting

This study was conducted at convalescent rehabilitation wards called Kaifukuki Rehabilitation Wards (KRWs). KRW is the system for subacute rehabilitation in Japan and is covered by governmental medical insurance. In KRW, patients undergo one-on-one rehabilitation with therapists up to 3 h every day. A typical schedule was 1 h in the morning and 1 or 2 h in the afternoon. Patients engage in self-training out of rehabilitation sessions if indicated. The content of the self-training is usually supervised by the therapist or doctor in charge. The participants can usually adjust the frequency and the intensity of self-training based on their own conditions and schedules. Interviews were conducted only once during the hospitalization and did not interrupt the participants' schedule of rehabilitation, meals, bathing, self-training, family visits, and meetings with doctors. It was mainly conducted in the afternoon between rehabilitation sessions and dinner. Interviews took place in the rehabilitation room or in the patient's room, where the content of the interview could not be overheard by others.

### Interviews

The interviewers were three occupational therapists (TY, MK, and SK) at the hospital with a mean clinical experience of 6.7 ± 1.5 years. They had no personal relationships with the interviewees. All the interview contents were recorded using an IC recorder with pre-obtained permission from the interviewees. The interview topic guide (Table 2) was developed based on discussions between the three occupational therapists (TY, MK, and SK) and a rehabilitation doctor (YO) with more than 20 years of clinical experience. All interviews were performed according to the interview topic guide. The term “motivation” in the study was defined as “the impetus to initiate, sustain, orientate, enliven, and move forward a goal-oriented action,” which was taken from the Japanese psychological dictionary (29). The definition was explained to participants prior to the interview to share a common understanding of the term. In addition, explanations for each question were prepared and shared among the interviewers; the meaning of the question was explained using these shared understandings prior to each question being asked and if the participant asked the interviewer about the meaning of the question. The interview was conducted by the occupational therapist who was not in charge of the patents' rehabilitation. Considering the psychological burden of the participants, the participants were told that the interview could be stopped immediately if a psychological burden arose during the interview.

**Table 2:** Interview topic guide.

1.	When does your motivation for rehabilitation become high (low)?
2.	What keeps your motivation high?
3.	What makes your motivation low?
4.	When your motivation becomes high (low), how does your behavior change?
5.	What is your motivation for rehabilitation?

### Analyses

Recorded interview data were transcribed to text format and analyzed using thematic analysis (24, 25). The transcripts were read thoroughly to acquire meanings from the words of each sample. Close readings of transcripts generated sentences as the record units. Record units of each interview were categorized into codes by their meanings. The codes were then categorized into subcategories. These subcategories were then categorized into categories, which were further categorized into core categories. An occupational therapist (TY) made record units and codes, and three occupational therapists (TY, MK, and SK) made subcategories, categories, and core categories through repeated discussion until a consensus was reached. The personally identifiable data were not included in data analysis to preserve anonymity. These procedures were conducted separately for samples of participants aged < 65 and ≥ 65 years to consider age-group differences, since lifestyles may differ between these age-groups. Comparisons of the characteristics including demographic variables and FIM scores were performed between aged and middle-aged groups with Mann–Whitney *U*-test and chi-square test depending on the types of variables. Statistical analyses were performed with IBM SPSS Statistics 27 (IBM Corp., Armonk, NY). *P* < 0.05 were considered statistically significant.

## Results

### Comparisons of Sample Characteristics Between Age-Groups

No significant differences were found in characteristics between age-groups, except for age and working status before onset (Table 1). Total FIM score ranged from 96 to 115 and 111 to 123 in aged and middle-aged groups, respectively. The participants in both age-groups had relatively high functional status.

### Codes and Categories

The average interview time for participants aged ≥ 65 was 19 min 46 s (*SD* ±193 s), while time for participants aged < 65 was 17 min 17 s (*SD* ±238 s). From interviews with participants aged < 65 years, 462 record units, 191 codes, 37 subcategories, 21 categories, and eight core categories were derived. From interviews with participants aged ≥ 65 years, 490 record units, 176 codes, 36 subcategories, 19 categories, and eight core categories were derived (Tables 3–**5**). Although some differences between the two age-groups were found in some categories and subcategories, the same eight core categories were derived for both age-groups. Among these eight core categories, seven included factors influencing motivation for rehabilitation and one category concerned the effects of motivation on patients' behaviors (Tables 3–**5**).

**Table 3:** Categories of personal factors derived from the thematic analysis.

**Core category**	**Category**	**Subcategory**	**Patients' narratives**
Patients' goals	Individual goals of activities and functions	Achievement goals pertaining to activities	“My goal is to recover my physical condition as soon as possible to get back to the same daily routine as before. Because of this, I can expend efforts on daily rehabilitation” (aged ≥ 65 years, Male 2).
		Goals for improvement of function and activity	“I want to be able to control my body freely again. Toward this end, I work hard to continue my rehabilitation” (aged < 65 years, Male 5).
	Role as a family member	Role as a housewife	“I hope that I will return home and regain my role as a housewife after discharge. When thinking about this goal, I understand what is necessary for me to do now” (aged < 65 years, Female 2).
		Role as a supporting family member	“Preparing meals has been my role. So, I am thinking now that I want to continue this role after discharged home” (aged ≥ 65 years, Female 1).
		Hope to acquire independence to reduce the burden on one's family^‡^	“If my walk is still unstable when I get discharged from hospital to home, it may be burdensome for my family. I don't want to bother my family” (aged ≥ 65 years, Male 7).
	Social participation	Hope to return to work	“My goal is returning to work. When I am thinking about that goal, I feel motivated and think that I should put much effort into my rehabilitation” (aged < 65 years, Male 7). “My goal is to return to my previous work. That is my motivation for rehabilitation” (aged ≥ 65 years, Male 5).
		Hobby	“I really like to play golf. I have to do training [to] play that” (aged ≥ 65 years, Male 3).
		Interaction with friends^†^	“I love to drink with friends. So, I have to be able to pick up a beer mug with my hand. To go to drink with friends again, I know that I should do rehabilitation” (aged < 65 years, Male 1).
	Discharge to home		“I want to go home and get back to my previous life as soon as possible. So, I should do what I have to do” (aged < 65 years, Male 6).
	Discrepancy between expected goal and reality^†^		“I had expected that I would have been able to walk without a cane by this time and would be preparing for the discharge by now. But the reality is not what I had expected; I am still using a wheelchair. The discrepancy between the hospitalization length I expected and reality makes me lose motivation” (aged < 65 years, Male 3).
Experiences of success and failure	Success experiences	Recovery from impairments and disability	“Motivation will be very high when I am able to do that [which] I couldn't do 1 week ago” (aged < 65 years, Male 3).
		Expansion of permitted range of activities in the hospital^‡^	“I want to expand my activity space. Then, I could go to a shop in the hospital, for example” (aged ≥ 65 years, Male 7).
	Failure experiences	Discrepancy between expectations and real performance of the tasks	“Sometimes I became disappointed that I couldn't do an easy task that I could do before” (aged ≥ 65 years, Male 3).
		Stagnation of the recovery^†^	“I was depressed when my movement was worse than it was yesterday” (aged < 65 years, Female 4).
		Repetitions of failure experiences^†^	“When I fail something repeatedly, [my] motivation [decreases]” (aged < 65 years, Male 6).
Physical condition and cognitive function	Physical condition	Fatigue	“I make an effort to do more when I have more physical strength; but I want to lie down when I am tired” (aged ≥ 65 years, Female 1).
		Physical weakness	“I lose weight when I can't eat my meals. Then, my physical strength decreases. In such cases, I don't feel like participating in rehabilitation” (aged ≥ 65 years, Female 1).
		Pain	“Motivation decreases when I can't do any daily rehabilitation training due to pain” (aged < 65 years, Male 3). “I'm worried that hard training may cause bad physical conditions such as muscle pain” (aged < 65 years, Male 1).
		Worsening of numbness^†^	“Some changes such as pain or numbness in my body reduced my motivation” (aged < 65 years, Male 9).
	Cognitive function^‡^		“I am here to do rehabilitation. We need to recognize why we are in this hospital. If I cannot understand the purpose of my training because of dementia, I cannot keep [my] motivation high” (aged ≥ 65 years, Female 10).
Resilience			“I recognize my disability. I need to live with this disability” (aged < 65 years, Male 8). “When a doctor said to me that I could have sequelae, I realized that I must make a tremendous effort for my own rehabilitation. I will be in trouble if I can't walk” (aged ≥ 65 years, Female 9).

† *Only for participants aged < 65 years*;

‡ *Only for participants aged ≥ 65 years*.

### Factors Influencing Motivation for Rehabilitation

The seven core categories for influencing factors of motivation for rehabilitation were patients' goals, experiences of success and failure, physical condition and cognitive function, resilience, influence of rehabilitation professionals, relationships between patients, and patients' supporters. The former four core categories were classified as personal factors (Table 3), and the latter three core categories were classified as social relationship factors (Table 4).

**Table 4:** Categories of social relationship factors derived from the thematic analysis.

**Core category**	**Category**	**Subcategory**	**Patients' narratives**
Influence of rehabilitation professionals	Relationships with therapists	Feedback from therapists	“In my opinion, whether or not there is a certain improvement for me is very vague. When the therapists give me positive feedback from their professional viewpoints, it makes me realize that I have improved somehow” (aged ≥ 65 years, Female 4).
		Reliance on therapists	“I trust therapists and medical staffs with my rehabilitation. These good relationships could increase my motivation” (aged ≥ 65 years, Male 7).
		Therapists' attitude toward patients	“My motivation for rehabilitation decreased when my therapists repeatedly pointed out my mistakes, which made me feel that I could not do anything” (aged < 65 years, Male 6). “When I wanted to engage in self-exercise for my weak points, the therapist kindly devised a regimen for me. Since then, I trusted the therapist more, and my motivation for rehabilitation has increased significantly” (aged ≥ 65 years, Male 3).
		Not influenced by phrases of praise^†^	“Sometimes, I would doubt the therapists' positive feedbacks. I was pleased to hear some of those praises; but I could not detect any truth in them” (aged < 65 years, Male 1).
		To do training for the sake of therapists^‡^	“My therapist has made such a great effort for me. I wanted therapists to feel that I was worth teaching. I think I need to rehearse what I have learned and engage in self-training” (aged ≥ 65 years, Male 2).
	Relationship with nurses	Feedback from nurses	“I think it is very important for me to receive positive comments from nurses such as ‘you have been improving and I know you are working hard on your rehabilitation’” (aged < 65 years, Female 10).
		Reliance on nurses	“I was very pleased when some nurses mentioned my improvement. That made me feel like they recognized my efforts” (aged < 65 years, female 10). “Some nurses are restless and that makes me hate them sometimes. I feel I am losing my motivation for rehabilitation when I am with them” (aged ≥ 65 years, Male 6).
	Relationship with medical doctors		“When a medical doctor in the acute hospital said that some impairments might remain after discharge, I was disappointed. But when another doctor in the same hospital said that they will make me improve within 2 months, that made me so motivated” (aged ≥ 65 years, Female 9).
	Exercise methods	Varied contents	“Exercises provided by my therapist are very good, of course, while other therapists would make me aware that other ideas for rehabilitation are also good” (aged < 65 years, Male 8).
		Shared goal setting and understanding of the purpose of the exercise	“I realized that becoming aware of the aim of the exercise is very important for increasing my motivation” (aged < 65 years, Male 9). “If goals are shared with therapists, motivation can be maintained even during hard training.” (aged ≥ 65 years, Male 8).
		Appropriateness of the exercise contents	“I feel frustrated if I am given the next level of exercise when I am yet to perfect the current level. I would be more motivated when the therapist clarifies my rehabilitation goals step-by-step and changes the exercise method accordingly” (aged < 65 years, Female 2). “In daily rehabilitation, therapists provide feedback on my weak points, which I also recognize, and teach me specific exercise methods to improve, which makes me motivated continuously” (aged ≥ 65 years, Male 5).
Relationship between patients	Observation of other patients	Observation of other patients' efforts	“Just by watching others making a big effort motivates me. The important thing is that he is making his best effort by his own way, which has nothing to do with the severity of the disability” (aged ≥ 65 years, Male 3).
		Observation of aged patients' efforts^†^	“When I see the effort of the aged patient, I feel that I should be making more effort on my own rehabilitation” (aged < 65 years, Male 9).
		Comparison with other patients' impairments and disabilities^‡^	“Sometimes I compare my abilities or differences to other patients. If other patients can do some movement, I want to be able to do those” (aged ≥ 65 years, Female 10).
		No influences from other patients	“All I can do is do my best. Other patients have no relationship to me” (aged ≥ 65 years, Female 9).
	Communication with other patients	Interaction with other patients	“An interaction with other patients is very important for me. It makes me feel like having a mutual encouragement” (aged < 65 years, Female 2).
		Conflicts with patients^‡^	“My motivation decreases when I have trouble in relationships with other patients” (aged ≥ 65 years, Female 4).
Patients' supporters	Existence of supporters	Family	“My family work so hard for me, so I must not complain about anything. I want to give back to them” (aged ≥ 65 years, Female 1).
		Coworkers^†^	“Some of my coworkers say they will wait for me to return to work. This is such a great motivation” (aged < 65 years, Male 9).
		Friends^‡^	“When my friends visit me, I really appreciate that. If no one comes, I will be depressed” (aged ≥ 65 years, Female 10).
	Relationship with supporters	Conversation with family	“My family member said to me that they were happy to see my abilities recovered, which makes me happy” (aged ≥ 65 years, Female 9).
		Encouragement^†^	“Keeping a high motivation is very tough. An encouragement from my friend helps me to maintain high motivation” (aged < 65 years, Female 10).
		Family visit^‡^	“When my family visits me, I get the feeling that I want to be discharged from here as soon as I improve significantly enough” (aged ≥ 65 years, Male 2).

† *Only for participants aged < 65 years*;

‡ *Only for participants aged ≥ 65 years*.

### Personal Factors

#### Patients' Goals

Regardless of age-group, participants mentioned that their rehabilitation goal regarding individual goals of activities and functions, role as a family member, social participation such as work and hobby, and discharge to home influenced their motivation. In addition, one participant (aged < 65 years) mentioned the discrepancy between expected goal and reality. A participant (aged ≥ 65 years) noted that acquiring independence to reduce the burden on his family was a key motivation. A participant (aged < 65 years) mentioned being motivated to interact with friends, which was included in the “social participation” category.

#### Experiences of Success and Failure

Recovery from impairments and disabilities was reported as experiences of successes. A participant (aged ≥ 65 years) reported that the ability to have an expanded range of activities at the hospital was a motivator. The discrepancy between movement that participants expected and real task performance was reported as a failure, which decreased patients' motivation. Some participants (aged < 65 years) mentioned that recovery stagnation and repeated failures decreased their motivation.

#### Physical Condition and Cognitive Function

Participants in both age-groups commented that physical conditions including fatigue, physical weakness, and pain influenced their motivation. Some participants (aged ≥ 65 years) reported that it was vital that they have cognitive function to understand the purpose of rehabilitation, and some participants (aged < 65 years) reported that worsening numbness influenced their motivation.

#### Resilience

“Resilience” refers to the ability to adjust and adapt to varied situations and overcome challenges after adversity, although no universal definition exists (30). Participants in both age-groups reported that high resiliency played a key role in maintaining their motivation.

### Social Relationship Factors

#### Influence of Rehabilitation Professionals

Relationships with professionals on the rehabilitation team—including therapists, nurses, and medical doctors—as well as exercise methods were classified into this core category.

Regardless of participants' age, they mentioned that professional positive feedback from therapists and nurses, reliance on therapists and nurses, and therapists' positive attitudes toward patients were motivating factors. Some participants (aged < 65 years) did not feel influenced by therapists' words, regardless of what they said; and some participants (aged ≥ 65 years) seemed to be motivated by their own beliefs—that they wanted to improve their body functions for the sake of their therapists. For some participants, doctors' explanations about medical conditions and prognosis also influenced their motivation. The explanation by a medical doctor that a patient could have sequelae and the percentage of improvement was lower than patient's expected reduced patients' motivation.

The variation and appropriateness of the exercises, sharing the goal setting, and understanding the purpose of the exercise positively influenced patients' motivation. Some advices and viewpoints for rehabilitation not only from the therapist in charge but also from other therapists could positively influence patients' motivation. Furthermore, some participants commented that motivation was positively influenced by the appropriateness of exercise contents, including adjusting the difficulty of the exercises, setting a goal through shared decision-making step-by-step, and changing the exercise regimen appropriately.

#### Relationships Between Patients

Observations of other patients' efforts influenced some participants' motivation, regardless of the severity of other patients' disabilities. Motivation was greatly enhanced by observing other patients completing their training; however, some participants were not influenced by other patients. Some participants (aged < 65 years) reported that observing aged patients' efforts was influential, while some participants (aged ≥ 65 years) reported that comparing their abilities to those of other patients had a positive influence on their motivation. Motivation increased when mutual encouragement was obtained. Some participants (aged ≥ 65 years) reported that motivation decreased owing to the frustrations from the conflicts with other patients.

#### Patients' Supporters

Existence of supporters and their relationships devised this core category. Regardless of age-group, participants reported that having a family and the conversations with them influenced their motivation. Regarding the existence of supporters, participants in both age-groups commented about their coworkers and friends. One participant (aged < 65 years) talked about getting encouragement from her supporters, and some participants (aged ≥ 65 years) reported the influence of family visits.

### Patients' Behavioral Change

The interviews revealed that patients' motivation had an effect on their behaviors in the hospital, although some participants reported that motivation had no influence on their behaviors (Table 5). Some participants reported on self-training and active participation for daily activities. Some other participants (aged ≥ 65 years) reported that they did not do self-training that made them tired when their motivation for rehabilitation was high because they wanted to display better performance during rehabilitation with therapists. For some participants (aged < 65 years), the frequency of communication with other patients and changes in facial expressions were also reported.

**Table 5:** Categories of effects on patients' behavior derived from the thematic analysis.

**Core category**	**Category**	**Subcategory**	**Patients' narratives**
Patients' behavior changes	Self-training	Extent of self-training	“When my motivation is high, I will walk through a corridor for self-training” (aged ≥ 65 years, Male 7).
		Cancelation of self-training for rehabilitation with therapists^‡^	“I never did self-training. If I did, I would be too tired to do rehabilitation with therapists” (aged ≥ 65 years, Female 1).
	Attitude	Attitude toward rehabilitation	“When my motivation is high, I demand my therapists to let me do harder training” (aged < 65 years, Male 6).
		Total time spent in a room	“I want to go shopping at the hospital store. When I have high motivation, I will not stay in my room” (aged < 65 years, Female 10).
		Attitude toward daily activities^‡^	“When my condition is good, I want to do what I can do in daily activities” (aged ≥ 65 years, Male 7).
	Expressions^†^	Facial expression	“I could smile when my motivation was high” (aged < 65 years, Female 2).
		Quality of expression	“When my motivation was high, I behaved cheerfully” (aged < 65 years, Female 2).
	Talking with other patients^†^		“I talked to others a lot when my motivation was high” (aged < 65 years, Female 10).
	No particular changes		“Motivation does not affect my activities. I just do what the therapist teaches me” (aged ≥ 65 years, Female 4).

† *Only for participants aged < 65 years*;

‡ *Only for participants aged ≥ 65 years*.

## Discussion

This study investigated the factors influencing subacute stoke patients' motivation for rehabilitation and the behavioral changes induced by motivation at a convalescent rehabilitation hospital. The strength of the study was that it comprehensively elucidated the factors influencing patients' motivation for rehabilitation with the qualified study methodology according to COREQ. In addition, this study revealed that behavioral changes due to motivation differ depending on patients' age. Seven factors influencing motivation were identified, all of which were categorized as extrinsic motivation. No factor included a purely intrinsic description such as enjoyment of rehabilitation itself. Regarding behavioral change, we found that high motivation does not always enhance activity in aged stroke patients.

As for the core categories influencing patients' motivation, four personal factors (patients' goals, experiences of success and failure, physical condition and cognitive function, and resilience) and three social relationship factors (influence of rehabilitation professionals, relationship between patients, and patients' supporters) were derived. While all these core categories are classified as extrinsic motivation, a category such as joy for rehabilitation itself (intrinsic motivation) was not derived from patients' narratives. The same core categories were derived from both aged and middle-aged patients. Therefore, these core categories will affect the majority of stroke patients' motivation for rehabilitation. Previous studies have shown that the motivation for rehabilitation in patients who have had strokes or various other diseases is influenced by individual traits (8, 9) and social environmental and social relationship factors (5–7, 10–13). However, these studies have not discussed the existence of intrinsic motivation for rehabilitation. Our results indicated that motivation for rehabilitation is mainly based on extrinsic, rather than intrinsic, motivation, at least for subacute stroke patients in convalescent rehabilitation hospitals.

The results indicate that, unlike sports or studies where internal motivation is vital, it is relatively effective to intervene with extrinsic motivation (1, 2), rather than intrinsic motivation, in the rehabilitation fields. Some of the personal and social relationship factors elucidated in this study are modifiable in rehabilitation practice, such as patients' goal setting and relationships with medical staff. Our results will be useful to help optimize rehabilitation practice and planning.

If the goal is set too high, the rewards the patients receive, such as functional improvement, will be less than what they expected. Such reward prediction errors could reduce patients' motivation to reach their goals (31). To maintain stroke patients' motivation, it is important to set appropriate goals and provide a variety of training content that can be tailored according to the patient's situation, such as age.

Previous studies pointed out the importance of relationships with others. For example, overprotection from medical staff and family makes patients incapable (9, 32), while appropriate information from rehabilitation professionals helps maintain patients' motivation (9). Our results are consistent with these studies and demonstrate the importance of social relationships, such as communication with medical staff, other patients, and family for maintaining the motivation for rehabilitation.

A previous qualitative study targeting medical professionals reported that motivation is affected by both physical and human environments, such as a well-maintained room and group treatment sessions (7). In our study, however, only human environmental factors such as medical staff, other patients, and supporters were derived, not physical environmental factors. The participants of this study were all patients who were admitted to the convalescent rehabilitation hospital for the first time and were not able to compare it with other environments, which could be the reason physical environmental factors were not derived.

Although no differences in the core categories were found based on patients' age-groups, some differences appeared in subcategories. In the middle-aged patients, factors associated with improvement in physical function were derived, such as stagnation of recovery, repetitions of failure experiences, and discrepancy between expectations and reality. In contrast, among the aged patients, the subcategory associated with self-care was derived, such as hoping to acquire independence to reduce the burden on their family. Younger participants with disability may have higher expectations of what they can achieve compared to individuals in other age-groups (33). During inpatient rehabilitation, aged patients' main goals were regaining independence in self-care activities and going home (34). In other words, factors related to physical function and its disability may affect motivation among middle-aged patients, while factors related to independent self-care and its disability may affect motivation among aged patients.

Regarding the behavioral changes induced by motivation, the frequency of self-training, attitude toward rehabilitation, and total time spent in a room were derived regardless of the patient's age. The majority of stroke patients in the hospital engage in self-training, rehabilitation, and activities of daily living more actively when they are highly motivated. Some aged patients, however, intentionally did not participate in self-training when their motivation was high to conserve their physical strength. Some of them wanted to show better performance during rehabilitation with therapists, to live up to their expectations. Meanwhile, the category related to expression and frequency of communication with other patients was derived in only middle-aged patients. Therefore, motivation is not likely to be expressed as an observable behavior such as emotional expression and self-training in some aged patients. Medical professionals tend to label patients' motivation based on observable activities such as demeanor and their compliance with rehabilitation (7). Our results indicate that if medical staff evaluate patients' motivation only from observational assessment, it may lead to mislabeling of motivation, especially in aged patients. Medical staff members need to understand that the differences in behavioral change are related to age. To assess patients' motivation for rehabilitation, it is desirable to evaluate their narrative or to use a validated evaluation scale for assessing patients' motivation.

There are several limitations in the study. Firstly, the sample size was relatively small, and the participants were enrolled with convenience sampling in a single institution. All the participants were Japanese and had relatively high functional level as measured with FIM. Since motivation may be influenced by the functional level as well as social factors, the generalizability of results obtained in this study should be done with caution. To examine the validity, a further study with a larger sample and participants with various functional levels using random sampling from multiple facilities in many countries should be conducted. Secondly, this study also involves the methodological limitations of qualitative studies. Although the study was conducted according to COREQ, the labeling could be different for another analyst. Some of the subcategories could be classified into multiple core categories. For example, hobby is classified as a subcategory in the category “social participation” but also could be allocated to the category “Individual goals of activities and functions.” Thus, interpretation of classification needs to be considered given the analysts' characteristics. Lastly, we cannot eliminate the possible existence of intrinsic motivation because we did not ask the direct/specific questions about the existence of intrinsic motivation, e.g., whether the participants enjoy rehabilitation itself. Although it is difficult to prove the non-existence of intrinsic motivation, more in-depth interview research will help clarify this point.

The motivation of patients with subacute stroke in convalescent rehabilitation hospitals was mainly influenced by factors related to extrinsic motivation. The patients' goals, experiences of success and failure, physical condition and cognitive function, resilience, relationships with rehabilitation professionals, and family and supporters were derived as the core categories. It is important that rehabilitation professionals consider these factors when helping patients stay motivated. Regarding behavioral changes induced by motivation, some aged patients do not show motivation via activities observable by rehabilitation professionals. For accurate evaluation of patients' motivation, rehabilitation professionals should use not only observation but also validated evaluation scales or patients' narratives. The findings of this study can help facilitate rehabilitation professionals' understanding of patients' motivation, encourage motivation, and develop optimal treatment plans.

## Data Availability Statement

The original contributions presented in the study are included in the article/Supplementary Material, further inquiries can be directed to the corresponding author.

## Ethics Statement

The protocol of this study was reviewed and approved by Ethic Committee of Tokyo Bay Rehabilitation Hospital and Ethic Committee of Waseda University. The patients provided their written informed consent to participate in this study.

## Author Contributions

TY, YO, and JY contributed to the conception, design, and methodology of this study. TY, YO, MK, SK, and JY performed the formal analysis. YO, RO, and JY supervised this study. TY wrote the first draft of the manuscript. TY, YO, RO, and JY reviewed and edited the manuscript. All authors contributed to manuscript revision, read, and approved the submitted version.

## Conflict of Interest

The authors declare that the research was conducted in the absence of any commercial or financial relationships that could be construed as a potential conflict of interest.
