# Supplementary material for: Motivation for Rehabilitation in Patients With Subacute Stroke: A Qualitative Study
Source: Front Rehabil Sci. 2021 Jun 7;2:664758. doi: 10.3389/fresc.2021.664758 (PMC9397769; doi:10.3389/fresc.2021.664758)
Supplement: Supplementary file 1 [file Data_Sheet_1.zip › data sheet/Sample characteristics.PDF]

Sample characteristics (aged < 65 years)

Sample no.	1	2	3	4	5	6	7	8	9	10
Gender, male/female	m	f	m	f	m	m	m	m	m	f
Age	49	62	57	51	42	63	59	55	49	48
Side of paresis (right/left/bilateral)	r	l	r	l	r	l	r	r	l	l
Days from stroke onset to interview	54	37	128	82	79	122	95	78	137	35
Days from admission to interview	29	15	97	46	35	67	58	62	106	18
Total hospitalization days	93	20	118	54	143	88	66	118	149	20
Number of family members living together	1	1	0	1	3	1	0	0	2	2
FIM† total score	117	126	117	124	84	110	121	118	93	125
FIM† motor score	83	91	83	89	52	82	86	84	64	90
MMSE‡	30	30	27	30	27	29	29	30	29	30
Living place before onset, home/not at home	home	home	home	home	home	home	home	home	home	home
Working status before stroke, Yes/No	Yes	No	Yes	Yes	Yes	Yes	Yes	Yes	Yes	Yes

†Functional Independence Measure; ‡Mini-Mental State Examination.

Sample characteristics (aged ≥ 65 years)

**Sample no.**	**1**	**2**	**3**	**4**	**5**	**6**	**7**	**8**	**9**	**10**
**Gender, male/female**	f	m	m	f	m	m	m	m	f	f
**Age**	83	75	75	71	81	80	84	78	77	76
**Side of paresis (right/left/bilateral)**	l	l	l	l	b	r	l	r	r	r
**Days from stroke onset to interview**	97	51	58	42	62	86	70	121	98	60
**Days from admission to interview**	73	27	32	28	30	44	36	102	59	36
**Total hospitalization days**	82	56	41	74	52	54	41	148	96	109
**Number of family members living together**	1	2	1	1	1	1	4	1	0	1
**FIM† total score**	116	119	118	110	109	93	87	92	113	108
**FIM† motor score**	81	84	84	82	79	68	70	63	83	72
**MMSE‡**	27	29	28	29	28	25	28	28	30	30
**Living place before onset, home/not at home**	home	home	home	home	home	home	home	home	home	home
**Working status before stroke, Yes/No**	No	Yes	No	No	Yes	No	No	No	Yes	No

†Functional Independence Measure; ‡Mini-Mental State Examination.
