# Supplementary material for: Motivation for Rehabilitation in Patients With Subacute Stroke: A Qualitative Study
Source: Front Rehabil Sci. 2021 Jun 7;2:664758. doi: 10.3389/fresc.2021.664758 (PMC9397769; doi:10.3389/fresc.2021.664758)
Supplement: Supplementary file 1 [file Data_Sheet_1.zip › data sheet/Coding of qualitative data in patients aged ΓëÑ 65(in Japanese).pdf]

S2 Appendix. Coding of qualitative data in patients aged ≥ 65

ID	Subject Number	Record unit	Code	Subcategory	Category	Core Category
PtE1	5	家に帰った後に必要な動作とかをやって言うときには，やらないとなって思ってやる気になりますよね．	生活に必要な動作が明確になることで向上する	活動目標からの要因	個人的達成目標	患者目標
PtE2	8	小さな目標でも何か持っている人は今リハビリでこういうことしようと考えていると思います．	目標達成がされていないため，やる気低下は経験していない			
PtE3	8	今までは少なくともないよね．				
PtE4	8	自分が目標とするところがあって，そこまでいってないものだから．				
PtE5	8	まだ途中経過だからね．				
PtE6	8	目標に向けて頑張っている間はモチベーションが落ちるってことはないよね．				
PtE7	8	良くなっているんだったらそれでいいっていう気持ちが大きい．	目標までのプロセスを理解していることで維持できる			

PtE8	8	自分の中で目標があるし，そこまでのプロセスがわかっているっていうのがあるよね．	
PtE9	2	目標があるからだよね．	病前身体機能獲得目標により維持できる
PtE10	2	それがあるからリハビリに苦もなく毎日出来ているも思います．	
PtE11	2	目標っていうのは，今までの健康な状態に体を戻したいっていうのが目標ですね．	
PtE12	2	手を動かしたいっていうことよりも元の生活に戻るっていうことが強いよね．	元の生活復帰目標にて維持できる
PtE13	5	同じような症状で入ってこられる方もいましたけど，年齢が高い方が入ってこられても汗をかく気がないんですよね．	病前生活再獲得目標により向上する
PtE14	5	そういうのをみていると「汗をかく気はないのか！？」って聞きたくなっちゃうんですよね．	
PtE15	5	そういう感じに漫然とリハビリを受けちゃいけないと思うんです．	
PtE16	5	自分から一生懸命にやって，リハビリをして自分で治そうっていう気持ちがあるかないかっていうのが大きいんじゃないかなって思うんです．	

PtE17	5	自分で自分の体を治す気はないのか！？って.	
PtE18	5	元のように戻したいって言う気持ちが一番ですよ.	
PtE19	5	そのために汗をかいてリハビリをして，教えてもらったことをやらないとダメだと思うんですよ.	
PtE20	9	早く体が，足も手も，自分で動かせるようにって.	早期退院と病前身体機能再獲得の目標がやる気を維持させる
PtE21	9	先生がいろいろと教えてくれて，これなら部屋でもできるかなってことやったりしています.	
PtE22	9	そうすることで早く家に帰れるかなとか思っています.	
PtE23	9	あとはそうすればはやく動けるようになるかなって思っています.	
PtE24	9	いつも，元の体に早く戻したいってことを考えているから，先生の言うことをしっかりやろうっていう気持ちがあります.	病前身体機能再獲得目標により維持
PtE25	2	やっぱり目標があるっていうことかな.	個人的目標の有無

PtE26	7	自分の体をもとに戻そうっていう思いが強いんじゃないのかな.	病前身体機能獲得目標により維持できる
PtE27	7	このままで歩くにも歩けないとかになるっていうのは嫌だから.	
PtE28	8	自分で何をやりたいのかっていうのを持たないとダメだよ.	自分自身の目標があることで維持できる
PtE29	8	モチベーションっていうか，それを自分でもっているか持っていないかっていうのが本当に大事だよ.	
PtE30	9	もしかして後遺症が残るかもっていうのがあったので，一生懸命やらないとなっていう思いですね.	病前身体機能再獲得目標で維持できる
PtE31	9	もとの体に戻りたいっていう目標があるかないかですよね.	
PtE32	9	目標がなくてただやっていると，もういいやって思っていることもあると思いますよね.	目標があることでやる気が維持できる
PtE33	9	もしかしたら目標がないともう帰っているかもしれませんよね.	
PtE34	2	目標があるからリハビリを前向きにやる気が出てくるっていうのが根本だよね.	病前生活獲得がモチベーション

PtE35	2	元の生活に戻るっていうことが目標だよ.	
PtE36	2	一日でも早く元の生活に戻れる状態にするっていうのが目標だよね.	
PtE37	6	元気な元の体になるためっていう目標のみですよ.	病前身体機能獲得がモチベーション
PtE38	8	正常に歩きたい.	病前身体機能に出来るだけ近づけたい
PtE39	8	正常な状態に戻りたい.	
PtE40	8	歩くのが多少遅くても，正常に近い状態で歩きたい.	
PtE41	8	手も十分に役に立たなくても，見栄え的に整っていればいい.	
PtE42	8	元に戻らないことはわかっているけど，そこにどれだけ近づけるかていう感じ.	
PtE43	8	家族のためっていうのは自分のためだから.	自分自身の目標達成がモチベーション

PtE44	8	周りからっていうのは，みんなは上がるとかいうけど，僕はそうは思わないんだよね．	個人的な目標の影響が大きい	
PtE45	8	みんなからだいぶ進んだとか，ほとんど出来るじゃないとか言われるけど，そういうのは全然気にしていない．		
PtE46	8	自分のペース，目標，それがいい方向にいっていれば他の人からの影響っていうのはない．		
PtE47	8	僕の目標はそこじゃないしね．		
PtE48	8	途中経過を評価されても，僕は全然関係ないね．		
PtE49	1	私はだいたい手ですよね．	上肢機能改善希望がモチベーション	機能能力改善目標
PtE50	1	手が病気になって動かしにくくなったんですよね．		
PtE51	1	元々家の仕事はやってなかったんですけど．		
PtE52	1	趣味として踊りとかもやっていたんだけどね，またみんなでやりたいなっていう気持ちがあるんだよね．		

PtE53	1	それを考えたときに，手を伸ばしたいなって思うよね．	
PtE54	1	手を動かしたいっていう動作の目標もありますし，	
PtE55	2	やる気が下がるってことは今までないね．	機能改善欲求により<br>やる気維持できる
PtE56	2	前の病院からもだけど，それはないかな．	
PtE57	2	一日一日をどうすれば進歩するかって考えているからね．	
PtE58	6	初めからここでお世話になったときから，俺はここで治すんだっていう目標がないとだめだよ．	身体機能改善目標に<br>より向上する
PtE59	6	治すっていう目標がないのに来ているようじゃダメだよ！	
PtE60	6	自分の体を自分で治すっていう気持ちが大切だよ．	
PtE61	5	早く良くなりたいっていう欲望ですかね．	身体機能改善欲求が<br>モチベーション

PtE62	5	体を良くするっていう気持ちですよ.			
PtE63	5	家族のためとか周りのためっていうよりも自分自身のためですよ.	機能改善目標がモチベーション		
PtE64	5	昔みたいに早足で歩いて，女房を置いてきぼりにしてやりたいっていうかね.			
PtE65	7	それはもう少しでも良くなろうっていう気持ちですよね.	身体機能改善欲求がモチベーション		
PtE66	9	早く元の体に戻したいっていう思いだけですよね.	早期に身体機能改善をしたい		
PtE67	9	それを糧に先生の指導を聞いて，周りの人の話を聞いて出来なかったらこうすればいいとかを聞いています.			
PtE68	1	それと，今ままでやっていた家事ね.	家事活動再開がモチベーション	主婦役割	家族としての役割
PtE69	1	洗濯とかを出来るようになりたいなっていうのもありますね.			
PtE70	1	まあ私は元々食事に関しては家事はしていなかったんですけどね.			

PtE71	1	洗濯はちゃんと今まで通りにしたいなっていう事がありますね.		
PtE72	1	あとは，田舎なんで，家の周りに苗木があるんですよね.	家庭内役割再獲得がモチベーション	家族支援役割
PtE73	1	そういうのを時期によって交換するっていうことをスムーズにやりたいっていうのもありますね.		
PtE74	1	なので，足っていうよりも手を治したいっていう気持ちが強かったですね.		
PtE75	1	苗木っていうのは，自分ちのやつで 10 何個もあるんですよね.		
PtE76	1	そういうのの植え替えなんかをスムーズにしたいっていう思いですかね.		
PtE77	6	家族の影響としては，女房が認知症なんですよ.	家族介護（家庭内役割）のため	
PtE78	6	女房が自分の事を必要としているわけですよ.		
PtE79	6	いつも腹の中では，女房のために先生から早く認めてもらって家に帰りたいっていう気持ちが有りますよね.		

PtE80	6	女房のためっていうのがリハビリのモチベーションをかーって盛り上がらせる要因になっていると思いますよ.		
PtE81	6	そのためにまずは体を良くしないといけませんからね.		
PtE82	6	元の生活っていうのも有りますよね.	家族介護の役割再獲得がモチベーション	
PtE83	6	女房がいて，支えていくっていう元の生活ですよね.		
PtE84	7	家族のためっていうのはあることはありますよね.	家族に迷惑をかけたくないという思いがモチベーション	介護不必要な生活の獲得
PtE85	7	よたよた歩いていても困るだろうし.		
PtE86	7	ある程度はしっかり歩けていないと困るだろうし.		
PtE87	7	家族に迷惑をかけたくないっていう気持ちもゼロではないですよね.		
PtE88	7	こういう状態になっちゃったからね.	家族に迷惑をかけないために身体機能を戻したいという思い	

PtE89	7	元の体に戻すっていうことと，家族に迷惑をかけないっていうことの２つでしょうかね.			
PtE90	2	あとは，家族に迷惑をかけたくないっていうのがあるよね.	家族への介護負担をかけたくないという思い		
PtE91	2	食べ物もそれにつながっているかもしれないけど，一人だけ刻みとかになると家族に迷惑がかかっちゃうからね.			
PtE92	2	動きもそうだけど, 階段とかもゆっくり杖ついて歩いているとかになると, 家族が見てないと行けないってなって迷惑がかかっちゃうよね.			
PtE93	2	お風呂に入っているときに見てなくちゃいけないとか.			
PtE94	2	健康なときは見てもらうってことはないんだから，その状態にまで戻りたいっていうのがあるよね.			
PtE95	5	私のケースだと仕事に戻りたいっていうのがあります.	復職目標により向上する	復職目標	社会的役割
PtE96	5	それが糧になってリハビリで汗を流しています.			
PtE97	5	仕事に戻りたいっていう目標に一心ですからね.	早期復職目標により向上する		

PtE98	5	仕事の人達にも迷惑をかけているわけですから.		
PtE99	5	その人達がいるし，その人達のためにも早く仕事に戻って頑張りたいなって思うんですよ.		
PtE100	5	治したいっていう信念を持っているかどうかです.	復職欲求により向上する	
PtE101	5	体もそうですけど，仕事についても戻るっていうことですよね.		
PtE102	1	仲間と踊りをしたいっていう大きな目標みたいなものがあるんですよね.	趣味活動再開がモチベーション	趣味活動再開
PtE103	3	私はですね，趣味は一つしかないんです.	趣味活動再開目標がモチベーション	
PtE104	3	趣味のゴルフをまたやりたいっていう思いが有りまして，それを目標にやってます.		
PtE105	3	あとやっぱりゴルフをしたいっていう目標もありますよね.	趣味活動再獲得がモチベーション	
PtE106	3	趣味があるっていうのは大事ですよね.		

PtE107	2	家に帰りたいっていう目標を再認識したときって言う感じというか.	自宅退院目標再認識で向上する		自宅退院目標	
PtE108	9	自分の体が大事だからなるべく先生の言うことを聞いて，痛くても我慢してやってきたんです.	早期退院を目標としてやる気を維持している			
PtE109	9	そうすれば早く退院できるかなって思って.				
PtE110	4	やっぱり自分が一日も早く出たいって言う気持ちでしょうね.	早期退院欲求により維持			
PtE111	4	出たいっていう目標.				
PtE112	4	一日も早くおうちに帰りたいっていう目標のため.	早期退院目標がモチベーション			
PtE113	4	家に帰って何するっていうこともないけど，とりあえず帰るっていうことかな.				
PtE114	4	子どもたちはとりあえず，トイレが自分で行けるようになればいいよって言ってくれてるし，他の事はどうにかなるよって言ってくれてるし，とりあえず家に帰れる状態になればいいかなって.				
PtE115	4	やっぱり一つ一つ効果が積み重なっていたなって感じるときかな.	改善実感により向上する	機能能力改善	成功体験	成功失敗体験の要因

PtE116	4	出来るっていう自信につながっていると思うしね．	
PtE117	4	日々教えていただいている療法士の先生の言うとおりに頑張ってやっています．	
PtE118	4	動作とか，体とかいろいろと良くなるっていうのはあるんですけど，両方共ありますよね．	
PtE119	8	授業で毎日自主トレの指導があれば，それが自分にあっていればやろうっていう気になるよね．	提示された自主トレメニューにより効果がでると向上する
PtE120	8	でも自主トレが成果が出続けないとダメなんだけどね．	
PtE121	8	一定の効果が出ればすごく良いよね．	
PtE122	8	でも逆の場合っていうか，自分が予想している数値と実際が違う場合には今の限りじゃないんだろうなって感じがして怖いよね．	改善継続がやる気維持には重要
PtE123	8	順調に良くなっているっていうのがそのための重要なことだよね．	
PtE124	10	自分自身も良くなっているっていう感じがしますよね．	改善実感により向上する

PtE125	10	最初は思ったところに手を動かすことが出来なかったりとか，手がしびれていたりとかありますけどね.	
PtE126	10	あとは家族が面談に来たときに「趣味はありますか」って聞かれたらしいんですけど，手芸とかやっていますって言ったら，続けたほうが良いですよって言われたんです.	自身の改善実感により向上する
PtE127	10	またまた家でやりかけていたのがあったので，今それをやっているんですけど，最初の頃は糸を引く力とかがうまく行かなくて，やっぱり前と全然違うなって感じていたんですけど，少しづつやっているうちに前に近づいてきたかなって感じますよね.	
PtE128	10	時間が経つごとに自分で出来ているっていうことがわかりやすいですよね.	
PtE129	1	その動きとかは実際に家でも必要なことですし，実際に出来たときっていうのは嬉しくてやる気になりましたよね.	動作改善実感により向上する
PtE130	1	入院した直後っていうのはできなかったことが出来るようになってきていた時期だったって言うこともあるでしょうね.	
PtE131	1	手に持てなかったものが，針でもなんでも持てたっていうのとか，日々の積み重ねが結果につながっているっていうのかな.	
PtE132	2	それに対してリハビリをして，その効果がどんどん上がるっていうのが重要だな.	改善実感継続によりやる気も維持する
PtE133	2	効果が毎日実感できるというか，良くなっていることが続くと下がりようがないよね.	

PtE134	3	自分の体が良くなったりした時とか.	身体機能改善によりやる気も向上する
PtE135	10	あとは良くなり続けているって言うこともありますし,	改善継続により維持
PtE136	2	流動食みたいなものばっかり食べていると気が滅入るよね.	食事形態の改善に伴いやる気も向上する
PtE137	2	そういうものばっかり食べていると筋肉もつかないしね.	
PtE138	2	こっちの病院に来ていろんなものを食べられるようになってきて筋肉がついたなって感じるよね.	
PtE139	2	食事自体もどんどん変わってきたなって感じはあるし，それはすごく嬉しいことだよね.	
PtE140	2	食事も生活に大きく影響するよね.	
PtE141	2	だから毎日美味しく全部頂いてますよ.	
PtE142	2	生活だけじゃなくてリハビリにもすごく影響すると思うよ.	食事形態の改善により全身状態が改善しやる気も向上する

PtE143	2	自分で美味しくいただければ，リハビリした時にこれが筋肉がつくようにつながっているかなとかっていうふうになる.	
PtE144	2	楽しみでもあるし，それ自体も成果が出てるっていう感覚があるからね.	
PtE145	2	食事は他の人と同等になったから，あ～ここまで来たんだなって感じがあるよね，効果が出ているだっていうか.	
PtE146	3	ある程度回復しているっていうことがだんだん分かるようになってくるんですよね.	日々の改善がモチベーション
PtE147	3	そしたら，もう少しやったらもっと良くなるんじゃないかっていう気持ちが湧いてくるんですよ.	
PtE148	3	毎日の回復を糧にリハビリをしているって感じですよね.	
PtE149	2	美味しいものが普通に食べたいっていうこともやる気につながっているよね.	食事が普通に取れることがモチベーションに繋がる
PtE150	2	今までは，刻んだ物とかとろみ剤が入っているものとかだったけど，それが固形物になってとろみ剤がなくなって，普通に売っているものが普通に食べられた時っていうのは，それはすごく美味しかったし嬉しかったよね.	
PtE151	2	市販のものを普通に食べられるっていうのはすごく嬉しいし，それ自体良くなったんだなっていう感じがするよね.	

PtE152	7	実際は，もう少し自由に歩ける範囲を増やしてほしいなっていう気持ちはありますよね．	活動許容範囲（安静度）を上げてもらえればやる気が向上する	活動範囲の拡大	
PtE153	7	まあ，それでも工夫して自分なりにやっているからね．			
PtE154	7	活動範囲が広がればなっていう欲求が湧くっていうのもあるかも知れませんね．	活動許容範囲（安静度）を上げてもらえればやる気が向上する		
PtE155	7	1 階に行って売店に行ってみたいとか．			
PtE156	7	やる気が無いとここだけでいいやって思っちゃうかもしれませんよね．			
PtE157	3	注意と言うか，集中力とかの課題で，なんでこんなことが出来ないのかなとか思うと落ち込みますね．	低難易度の課題失敗による落ち込み	実動作と期待した動作の差	失敗体験
PtE158	3	体のことでも，いくら意識してもその通りに動かすことが出来ないってなると落ち込むこともあるよね．			
PtE159	3	元はそんな簡単なことだったら出来たのにっていう失敗体験と言うか．			
PtE160	3	そういうのがあると落ち込みますよね．			

PtE161	1	転んだときとかも，それから起き上がるにはどうしたらいいか教えてもらわなくちゃならないんだから，やる気が落ちるってことはなかたですけどね.	失敗体験から学ぶことでやる気が上がる			
PtE162	1	転んだっていうことをマイナスじゃなくて，プラスにとらえてね.				
PtE163	1	もう少し体力があればもっと張り切ってやれるのになって感じますよね.	身体的疲労が溜まっている時には低下する	身体的疲労	身体状況	心身状況からの要因
PtE164	1	疲れがあるときは何をしても伸びちゃいますし，横になりたくなりますよね.				
PtE165	1	私自身はいつも気はあるんですけどね.	身体的疲労感により停滞する			
PtE166	1	体力が伴わないとかでやりたくないなって思うこともありましたね.				
PtE167	1	でも一旦出ちゃえばその気になることも有りますけどね.				
PtE168	1	自分で今日は一時間が無理だなって思ったときには担当の人に伝えたりして希望を言いました.				
PtE169	1	やる気が無い時って言うと，朝から力が出なかったね.	身体的疲労感により停滞する			

PtE170	1	朝から寝ていることもあったね.		
PtE171	4	でもたまにぶすっとしている時って言うのは疲れているときかな.	身体的疲労感により停滞する	
PtE172	4	疲れてくると喋りたくなかったり，動きたくなかったりするよ.		
PtE173	5	やる気が無い時って言うのは疲れているときが多いと思うので，寝ていることが多いですかね.	疲労感が強い時は低く，臥床傾向になる	
PtE174	10	疲れを残さないようにリハビリをするっていうのは大切ですよね.	疲労感が残るとやる気にも影響する	
PtE175	10	朝とか起きたときに大丈夫かなって感じることもありますしね.		
PtE176	1	下がることって言うのは，食事が取れないっていうことが有りますね.	食事がとれないことにより低下した	身体的虚弱
PtE177	1	元々しょっぱいものばかりを食べていたもので.		
PtE178	1	病院食は非常に体によく出来ているでしょ.		

PtE179	1	全然とれなくて，栄養のあるゼリーとかも配布してくれたんですけど，それでもダメで.		
PtE180	1	そんなこんなしているうちに体重がかなり減ってしまって.		
PtE181	1	食べられないと，体力がなくなって，リハビリも行きたくないなって感じますね.		
PtE182	9	やっぱり自分で歩けなくなったりっていうのもあるかもしれないけど，あっちが痛いこっちが痛いっていう痛みですよね.	疼痛の出現はやる気を阻害する	疼痛
PtE183	9	そういうのがあると部屋にこもっちゃったりして，もうリハビリしたくないって言っている人もいますよね.		
PtE184	9	元々の病気以外の痛みとかですよね.		
PtE185	9	リハビリをしたいと思っていても，痛くて出来ないってなるとだんだんとやりたくなくなりますよね.		
PtE186	9	まずはリハビリよりも痛みをとることだなって感じになりますよね.		
PtE187	10	リハビリをするためにこの病院に来ているので.	リハビリ病院入院の意味理解により維持	認知機能状況

PtE188	10	ちゃんと歩けるようになって帰らないと意味が無いので.		
PtE189	7	とにかくリハビリをしないとっていう考えになっているから.	リハビリ病院入院意味理解により維持できる	
PtE190	7	ここに入院した時点でリハビリをしないといけないっていう考えになりますよね.		
PtE191	10	やらないといけないっていう気持ちがありますしね.	リハビリ病院入院の意味理解により維持できる	
PtE192	10	リハビリをするためにここに来ているんですからね.		
PtE193	10	ここにいる意味を理解していないとか.	リハビリ病院入院目的理解がないと停滞する	
PtE194	10	リハビリをしに来ているんだって思わないとダメだと思います.		
PtE195	9	私って病気っていう病気はしたことがないからやるだけやれば良くなるだろうって考えちゃうっていうのもありますよね.	病気に対してのレジリエンスな捉え方	障害のリジリエンス
PtE196	9	一番最初に入ったときに，もしかしたら後遺症が残るかも知れないっていうのも聞いていましたから，それが逆に頑張らないとって思うようになりましたよね.	後遺症に対してのリジリエンスな考え方により向上する	

PtE197	9	歩けなくちゃ困るって思って頑張ろうって.				
PtE198	9	下がるっていうことはなかったですよ.				
PtE199	9	今日は何やりましょう？とかって聞いて，元の状態に戻りたいって思うので.				
PtE200	9	疲れた時って言うのも，私自身丈夫なので，それでやる気が下がるっていうのもなかったです.				
PtE201	3	やる気がさがるときっていうのは，療法士の指導者の先生が良く出来たって褒めてくれたときには，やる気が出るんですけど，そうじゃない時って言うのもあるのでそういうときにはちょっと落ち込みますよね.	療法士からのポジティブフィードバックにより向上する	療法士からのフィードバック要因	療法士との関係性	専門職からの要因
PtE202	3	なので，出来なくて落ち込んでいるときにも大丈夫とか，よく出来たねって言われるとやる気があがりますよね.				
PtE203	3	特に訓練中とかで出来ないことがあると落ち込んじゃったりしましたよね.				
PtE204	4	だから効果が出ているのか，わからないというか曖昧というか.	療法士からの改善フィードバックにより実感でき，向上する			
PtE205	4	でもそこを療法士の人がみて，がんばってるね，良くなってるねとか声かけてくれると，良くなってるのかなって.				

PtE206	6	担当の療法士は本当に褒め上手だからね.	療法士からのポジティブ・フィードバックにより向上する
PtE207	6	でも，こき使うときはすんごいんだよ.	
PtE208	6	そうやっていても後で褒めてくれるし，そのカバーがうまいんだよね.	
PtE209	6	早く良くしてくれようとしているのがすごくわかっているからね.	
PtE210	9	やっぱり少しでも療法士の先生から褒められるっていうときにはやったってなりますよね.	療法士からの褒めにより向上する
PtE211	2	先生方みんなに斎藤さん今日は良くなってないなとかってなればやる気は下がるかもしれないけど.	改善点を療法士からフィードバックされることで向上する
PtE212	2	まあ，先生たちは言わないだろうけど.	
PtE213	2	先生が休みだった後とかに，大分良くなっているねとか言われるとうれしいよね.	
PtE214	2	自分の感覚で良くなっているっていってもわからないからね.	療法士からの改善のフィードバックが重要

PtE215	2	数値として前よりも良くなっているって言われたほうが嬉しいよね．	
PtE216	2	周りからの励ましって言うと，先生からの褒めが一番の励ましだよね．	療法士からの改善点の具体的なフィードバックで向上する
PtE217	2	具体的に言われたほうがいいよね．	
PtE218	2	周りから良くなってるねとか言葉でいられるのは，いくらでも言えるからね．	
PtE219	2	実際に歩ける距離が伸びたとか数値が出たほうが良いよね．	
PtE220	5	自分自身が感じている問題点の解決策を早く見つけたいっていう思いがあるんでしょうね．	患者自身の問題点を指摘されることで向上する
PtE221	5	どうすればいいのかっていうのは，自分自身じゃわからないですからね．	
PtE222	7	家族からって言うと，よくやれって言われますけどね．	患者自身の問題点を指摘されることで向上する
PtE223	7	家族としては全然分かっていないと思いますからね．	

PtE224	7	リハビリの内容はよく分かっていないですけど，ただ頑張ってっていう感じですかね.	
PtE225	7	リハビリやっている中で，どれが難しいかっていうのはよくわからないね.	
PtE226	7	けど，物によっては体がきついなって感じるときとかは，少しだけど感じたりもするかな.	
PtE227	7	そういうものもだんだんと慣れてきたりすると訓練もっとやろうっていう気持ちになりますよね.	
PtE228	6	あとは担当療法士の方から褒められているっていう感覚があることかな.	療法士からの褒めにより向上する
PtE229	6	褒められるっていうのは嬉しいからね.	
PtE230	8	日々改善しているかっていうのはわからないんだよね.	療法士からのフィードバックが影響する
PtE231	8	療法士から言われたことがほぼないからね.	
PtE232	8	今までよりは良くなったとかは言われるけど，今の目標に対して何％だっていうのは言われたことがない.	

PtE233	8	でも目標が大きいから，簡単に何%って言われたくもない気持ちもあるかな．	
PtE234	10	それを周りが認めてくれているって言う事ですかね．	周囲からの改善承認により維持
PtE235	4	周りからの影響っていうのももちろん有りますよ．	療法士からの声かけにより向上する
PtE236	4	みんなリハビリとかで歩いていると，療法士の方で頑張ってとか言われるとやる気になりますよね．	
PtE237	4	全然知らない人とかからでも，声かけられると励みになりますよね．	他者からの声かけにより向上する
PtE238	4	自分自身は全然知らない人でも，声をかけてくれると，みててくれているだって感じがしてすごく良いですよね．	療法士からの声かけで向上する
PtE239	4	周りの患者さんとの関わりっていうよりも，療法士の人との関わりが大きいですよね．	
PtE240	1	でも寝ていても療法士が来て，あれやれこれやれっていうからね．	療法士の促しによりやる気が向上する
PtE241	1	そうするとやっちゃうんだよね．	

PtE242	6	先生たちからの影響は凄く大きいんじゃないんですかね.	療法士との交流により維持向上	療法士への信頼感
PtE243	6	コミュニケーションっていうか先生たちと話し合いっていうのは大切なんですよ.		
PtE244	7	スタッフがね，非常に良かったと思いますよね.	療法士との良好な関係性により向上する	
PtE245	7	付き合いというか，全てにおいて.		
PtE246	7	スタッフっていうのはリハビリの担当者ですよね.		
PtE247	7	ですけど，代行者も含めて全体的によくやってくれていたと思いますよ.		
PtE248	7	退院が近いですけど，まあまあうまく行っていうとおもいますよ.	医療スタッフへの信頼関係があることでやる気が維持できる	
PtE249	7	そりゃ前の状態と比べると違うかもしれないけどね.		
PtE250	7	けど，とりあえずスタッフの人達の指示に従っているっていうのが大きいですね.		

PtE251	7	スタッフに任せておけば大丈夫っていう信頼感って言うものがあってのことですしね.		
PtE252	2	皆さん先生方は仕事っていってもひとりひとりの為を思って指導してくれているから，心から感謝するしやらないとなって気持ちになるよね.	担当療法士への恩返しのために訓練を行っている	担当療法士への恩返し
PtE253	2	恩返しじゃないし，お礼じゃないけど，ありがたいなとかやらないとな，元気にならないとなっていう気持ちもあるよね.		
PtE254	2	せっかくリハビリやって教えてもらっているから，その先生たちのためにも教えた甲斐があったっていうふうに感じてもらうというか.	担当療法士のために訓練を行っている	
PtE255	2	そのためには，自主トレとかをベッドでやってみたり，今日そういえばこういうことを教わったなとか確認してみたりそういうことが必要だと思いますよ.		
PtE256	7	訓練は自分のためなんですけど，療法士のためにっていう側面も確かにあるかもしれませんね.	療法士のために成果を出したいという思いが向上させる	
PtE257	7	その患者が良くなって，その喜びを感じてもらっていると思うので.		
PtE258	7	そちらの熱意に応えないとっていうことも多少はありましたよね.		
PtE259	7	義務とかそういうのではなくて，ただ純粋に良くしたいんだなっていう気持ちが伝わって来ますからね.		

PtE260	7	よくやってくれているなって感謝していますよ.			
PtE261	3	周りからの影響っていうのも有りましたね.	療法士の患者に対する積極的態度により向上する	療法士の患者に対する態度	
PtE262	3	私がやりたいっていうことを理学療法士さんとか作業療法士さんとか一生懸命考えてくれましたね.			
PtE263	3	それで自分の欠点もあるんですけど，それに関して自主トレをしようって考えたときに，自主トレ表を作ってくれて，これを信じてというか，療法士の方を信じてやろうっていう気持ちでやる気が出ましたよね.			
PtE264	3	励まされるっていうよりも，療法士の先生とか看護師さんが丁寧に接してくれるので頑張ろうっていう気持ちにはなりますよね.			
PtE265	6	看護師さんからも良くなったねって言われることはうれしいですよ.	看護師からのポジティブフィードバックにより向上する	看護師からのフィードバック要因	看護師との関係性
PtE266	6	自分の努力を認めてくれているっていうか本当に嬉しいんですよ.			
PtE267	3	療法士の先生もそうですけど，看護師さんでも声をかけてもらえると嬉しかったですよね.	医療者との良好な関係性構築により向上する	看護師への信頼感	
PtE268	3	患者さんでも療法士さんでも看護師さんでも良い関係性っていうのはとても大事だと思います.			

PtE269	6	担当してくれている人とのやり取りに尽きると思いますよ．	看護師との良好な交流により維持できる		
PtE270	6	話していると落ち着きがないとかっていう人に対しては本音は言わないよね．			
PtE271	6	看護師さんによっては，なにくそって思うこともあるけど，そういう人と接しているとやる気っていうのは出てこないよね．			
PtE272	9	最初はお医者さんから後遺症があるかもしれないって言われてたけど，今まで健康だったし歩けなくなるのは困るなって思っていたんです．	医師からの目標達成までの期間提示により向上する		医師との関係性
PtE273	9	この病院に入る前に院長先生から 2 ヶ月半で戻してみせますっていうのを言ってくれたから，それもあって頑張ろうって気持ちが高くなりましたよね．			
PtE274	3	今まではそれに向かって我流で頑張ってやってきていたんですけど，ここに来て理論的でね，体を触っていただいて，ここがこうでこうでって教えていただいて．	適切な訓練方法提示により向上する	訓練内容の適切さ	訓練方法
PtE275	3	こういうやり方であればもっと良くなりそうだなって感じてやる気が湧きますよね．			
PtE276	7	訓練メニューとか内容とかていうのはこっちは全然わからないからね．	療法士との訓練メニューが円滑に進行することで向上する		
PtE277	7	こちらとしては，スタッフの人が持ってきたものをやっているっていう感じですよ．			

PtE278	7	そういう訓練のやり取りとかが円滑に行えているっていうんも大切ですよね.	
PtE279	8	あとは，この手があるでしょ？	患者へのオーダーメイドな支援提示により向上する
PtE280	8	そのために，他の患者さんには悪いかもしれないけど，私の手に合致した内容の物を提供してくれれば上がるよね.	
PtE281	8	自主トレメニューとか.	
PtE282	5	日々訓練をしていて，姿勢を直せとかいろいろと指導をしてくださるっていうのが大きいように感じますよね	療法士の指導内容が的確であることで向上する
PtE283	5	自分が弱いなって感じているところを言ってくれて，それに対する方法とかを指導してくれるとやろうっていう気持ちが続きますよね.	
PtE284	8	1 つはリハビリの厳しさっていうか内容の問題.	訓練難易度調整不備により停滞
PtE285	1	でも訓練内容の調整とかの希望は言うようにしているし，実際に調整してくれますしね.	身体状況に応じた訓練強度の調整により向上する
PtE286	1	調整してくれるっていうことは私にとってとてもいいことだったかな.	

PtE287	9	そういうタイミングで先生からも宿題みたいなものがあるっていうのもいいですよね．	自主トレメニュー作成がやる気につながる	
PtE288	9	口のリハビリとかでも体のリハビリとかでも，歳だから難しいのは出来ないですけど，出来るものはありますよね．		
PtE289	8	90 以上のじいさんにハードな目標はだめでしょ．	患者それぞれに適した目標設定により維持できる	
PtE290	8	10 年かかる目標とかも提示してもだめでしょ．		
PtE291	8	目標を持たされるようなガイダンス．	療法士と患者の目標共有により向上する	療法士と患者の訓練目標共有
PtE292	8	それがあって，目標が提示されて，それが自分が思っているものと合致すれば上がる．		
PtE293	8	自分の目標と，療法士の目標って大体合致してるけどね．	療法士と患者の目標共有にて向上する	
PtE294	8	そうじゃないとおかしいじゃない？		
PtE295	8	大体は合致するし，そういうふうに持っていかないとダメだし，大体はもっていっていると思うけどね．		

PtE296	8	目標共有がなされるっていうのは大きいよね.		
PtE297	8	訓練の厳しさっていうのは，自分にそれがあっているかどうかっていうのが問題.	目標が療法士と患者間で共有されていると維持する	
PtE298	8	目標が共有されているかっていうのに繋がる.		
PtE299	8	患者とインストラクターとの関係の問題だよね.		
PtE300	8	訓練とかでも担当じゃなくて他の人が来て，いきなりハードなことをやることもあるでしょ？	代行担当者のいつもとは違った訓練内容により向上する	訓練バリエーションの豊富さ
PtE301	8	階段とかいきなり杖無しでやってみてとかね.		
PtE302	8	でもそれはそれで嬉しかったよ.		
PtE303	8	ルーティーンじゃなくて，こういう目標でこれをやりますとか，しっかり目標があって違うことをやるっていうのはすごく良いよね.	訓練バリエーションの豊富さにより向上する	
PtE304	8	一人ひとりが私のために考えた場合，どういうことをやるのっていう.		

PtE305	8	バリエーションがあったほうが良いよね.				
PtE306	8	目標が一緒で，練習するレベルはだいたい一緒だと思うんですよ.	同目的の訓練バリエーションが多いと向上する			
PtE307	8	色が少し違ってるんだけどね.				
PtE308	8	でも僕はそっちのほうがすごく良いと思うんだよね.				
PtE309	4	今まで全然運動してきてなかったから訓練自体はすごく楽しいんです.	訓練自体を楽しく感じている			
PtE310	2	スケジュールが空いちゃうときには退屈だなって感じるよね.	訓練スケジュールからの影響			
PtE311	10	他の人を見て早く追いつきたいなって感じることもありますよね.	身近な能力が高い他患との比較により向上する	他患と自身の能力比較	他患観察からの要因	患者関係からの要因
PtE312	10	後をついて退院したいなって感じることもありますよね.				
PtE313	10	他の人とリハビリの進度を比較したりていうのも少しはありますよね.				

PtE314	10	そういうのはやっぱり食事で一緒の人とかが多いですよね.		
PtE315	10	いいなって励みになるっていう対象だと，少し自分よりも出来る方が多いですよね.		
PtE316	1	他の人よりも良くなりたいっていうのは多少なりとも有りましたよね.	他患との競争により退院へのモチベーションが向上する	
PtE317	1	みなさんが良くなって退院していけば，私もそうしたいっていう感じでね.		
PtE318	1	一緒に食事とかをしている人が，退院するとかなると，私も早く退院したいなっていう気持ちになりますよね.		
PtE319	2	患者さんと比べるというかそういうのは，患者さんは各々違うんだから，症状も違うから，それと比較してあの人よりもっていうのはないね.	患者間比較はない	他患からの影響はない
PtE320	4	周りの方と比べてって言うことは全く無いですね.	他者比較はない	
PtE321	4	やっぱり自分との戦いだと思うんです.		
PtE322	4	周りとの関わりっていうと疲れることもあるからあまりしゃべらないようにしているんです.		

PtE323	7	他の患者と比べてっていうのは特にないですよね．	他患との比較は行わない
PtE324	7	ただ，リハビリの時間だって感じてただやるっていう程度だと思いますよ．	
PtE325	8	他の患者よりもっていうのもまったくない．	他患とは状態が違うため，比較をしても意味がない
PtE326	8	だって人間ってバラッバラでしょう？	
PtE327	8	似たような物を持っていても違うじゃない？	
PtE328	8	例えば，脳卒中で私もこうなりましたっていっても，結果的に違うんだよ．	
PtE329	8	病気として一つの括りで判断されるけど，全然一緒じゃないんだよね．	
PtE330	8	この人は，こうっていう評価があるかもしれないけど，この人は８０％，この人は何％っていうのがあっても良いんじゃない？？	
PtE331	8	自分の進路さえ捕まえていればいいと思うけどね．	

PtE332	9	周りの人を見てリハビリ頑張るっていうよりも，自分のためって言うほう大切ですよね．	他者からの影響よりはない（自分でマネジメント出来ている）	
PtE333	9	人は人だから，あまり関係ないですよね．		
PtE334	9	周りの人が頑張っているのを見ると，頑張っているんだなって思うんですけど，自分は自分で頑張るっていう思いが強いですよね．		
PtE335	5	励ましとか周りからの影響っていうのは私のケースではなかったですね．	他者からの声かけは影響しない	
PtE336	5	もう自分で自分をよくしようっていう気持ちでいっぱいでしたから．		
PtE337	3	他の患者さんとの競争とかっていうのは私は全く無いですね．	他者の頑張っている姿を見ることからの自身の行動変容	他者の努力を見ることの要因
PtE338	3	頑張っているなあの人とかっていうのは，あるし，競争じゃなくてそういう人を見ていると自分も頑張らないとなって感じにはなりますよね．		
PtE339	3	頑張っているなって感じる相手とかは，動ける動けないとかっていうのは全く関係なくて，とにかく頑張っていると言うか，そういう人を見ると自分もってなりますよ．		
PtE340	4	周り見ていてみんなが歩いて退院するからそういうのを見ていると励みになりますよね．	他者改善を見聞きすることでやる気が上がる	

PtE341	4	先に入院している方々のリハビリで，これまでのことを聞いて励みにしています.	
PtE342	5	あの人が頑張っているから自分も頑張ろうっていう気持ちは有りましたよね.	他者の頑張りを見ることによる行動変容
PtE343	5	見ていて同じような状況の人とかが頑張っているもを見ると，あの人に遅れを取らないように頑張らないとっていう気にはなりますよね.	
PtE344	5	そういう気持ちはプラスの方向に働いていると思うんですよね.	
PtE345	5	そういう人たちっていうのは私と同じぐらいの年齢の方っていうのがあると思いますね.	同年代患者（高齢者）の頑張りを見ることによる感化
PtE346	5	練習している内容が同じような感じとかですね.	
PtE347	5	その人が自分が出来ない動きとかをやっているのを見ると，私も頑張ろうっていう気持ちになりますよね.	
PtE348	5	若い人を見てっていうのはないですし，違う状況の方とかだとそういうのは起こりにくいですよね.	
PtE349	6	他の人から影響を受けるって言うと，みんな状況は違いますが，みんなそれぞれで頑張っていますからね.	他者の頑張りを見ることにより感化される

PtE350	6	そういう姿を見て自分も頑張らないとって感じることはありますよね.			
PtE351	10	杖をつかないで歩けるようになった方とか，自分よりも動ける状態で退院が決まった方とか.	自身より高いゴールで退院した他患の存在で向上する		
PtE352	10	そういう方を見ると自分も早く退院したいなって感じたりしますよね.			
PtE353	3	患者さん同士って言うと，同室の方がとても明るい方たちで良かったなって感じますよね.	他患交流により向上する	他者との交流による要因	患者交流による要因
PtE354	3	最初個室に入っていたんですけど，最初は出るのが嫌だったんですよ.			
PtE355	3	でも結局出てよかったと思います.			
PtE356	3	っていうのは前野病院からずっと人との会話と言うか関わりがなかったんで，人恋しかったっていうのもあるんでしょうね.			
PtE357	3	部屋から出てうろうろしていて，他の人と話せたときにはすごく嬉しいって感じがしましたね.			
PtE358	9	ご飯も食べれていますし.	食事同席患者との交流により向上する		

PtE359	9	ご飯も一緒にしているといろんな話をするじゃないですか，それも楽しいですよね.	
PtE360	9	部屋で食事やるよりはみんなでやったほうが楽しいですよね.	
PtE361	9	私も最初は部屋で食べたいって言っていたんですけど，他の人と交流するっていうのはすごく大事ですよ.	
PtE362	9	リハビリのことだけじゃなくて，個人的なこととか家庭のこととかも話しますしね.	他患との交流により向上する
PtE363	9	家の事でこうだよとか他愛もないことっていうのが話せるじゃないですか.	
PtE364	9	リハビリのことっていうよりも他の人ととにかく交流するっていうのも大事ですよね.	
PtE365	10	周りからの影響っていうのも大きいですよね.	他患との交流により向上する
PtE366	10	患者さんとの関係だと，食事を一緒にしている方とかだと，皆さん元気だしお話していても楽しくさせていただいているので，短い間ですけど，その会話でお互い励みになっていると思います.	
PtE367	10	内容としてはリハビリのこともありますけど，それ以外の身の上話とか，昔の話とかもします.	

PtE368	10	リハビリの話だけって言うよりもざっくばらんに交流するって言う感じですね.		
PtE369	10	部屋にいて一人でいるよりも他の方と喋っている方がいいですよね.		
PtE370	10	患者さんというかそういう交流っていうのは本当に励みになりますよね.		
PtE371	2	やる気のない方っていうのは良くわからないね.	同室患者のモチベーションが高いことで自身も維持できる	
PtE372	2	部屋の人もみんなやる気がある人達だから，それが普通になっちゃってるかもしれないけど.		
PtE373	4	下がる時って言うのは，病院での生活で人間関係の悩みとか.	入院患者同士のトラブルに巻き込まれるとやる気が下がる	患者間トラブルによる要因
PtE374	4	病院内とか病室内とかネガティブな発言というかそういうのを聞くとすごくテンションが下がる.		
PtE375	4	私はよくわからないんだけど，患者さん同士で，あの人は特別なのよとか，看護師さんが対応が違うとか，なんかそういうことを話されると私巻き込まれてるって感じがする.		
PtE376	4	私自身は全然そんなの感じてないんですけど，その人特有の感じ方と言うか，そういうのを聞くとすごくテンションが下がる.		

PtE377	4	患者さん同士の些細なやり取りというか，違いというか．	
PtE378	4	そういうのを聞くと，一刻も早くこの病院から出たいって思う．	
PtE379	4	リハビリ自体っていうよりも部屋でのゴタゴタとか耳にすると早く出たいなって感じるよね．	
PtE380	6	部屋の人達もみんないい人ですよ．	患者同士の人間関係の不和によりモチベーションが影響する
PtE381	6	でもね，なかにはくそって思う人もいるんですよ．	
PtE382	6	でもね，それ以上に蜜に接してくれている先生たちの関わり方が大きいと思うんですよ．	
PtE383	6	くそって思う人と関わっているとダメだと思いますよ．	
PtE384	4	病院に入っていろいろな事を感じたから（患者同士のトラブル），ここに長くいちゃいけないって感じてるんです．	患者間トラブルの発生によりやる気が停滞する
PtE385	4	女性独特なんでしょうけど，弱い人を助けて上げたくなると思うんですけど，ついつい自分も病人だしっていう感じになるんでしょうね．	

PtE386	4	そんなトラブルに巻き込まれて，ストレスになっちゃうとこっちが体調崩しちゃうって思う.				
PtE387	4	最近では，いびきで眠れないから薬貰って寝てたりするんですけど，いびきかいている人に対して「嫌がらせ」って思っちゃったりもしている人もいるんですよね.				
PtE388	6	家族というか女房の存在ですよね.	家族の存在により向上	家族の存在	支援者の存在の要因	支援者からの要因
PtE389	2	でも，家族が来ているっていうときにも，前よりも良くなってないと見せたくないなとか会いたくないなっていうこともあるかもね.	家族に改善したところを見てほしいという思いで向上する			
PtE390	2	良くなっていると見てくれっていう感じにもなるけど.				
PtE391	2	まあ，来てくれるだけで嬉しいんだけどね.				
PtE392	2	家族が来てくれていないと孤独な戦いになるからね.				
PtE393	1	糧っていうのはやっぱり家族の存在ですかね.	家族への恩返しがモチベーションになっている			
PtE394	1	家族も私がここに来るまでに相当な努力をしてくれているんですよね.				

PtE395	1	退院までの準備とか，入院中の調整とか，お見舞いとか.		
PtE396	1	そういうのに恩返しっていうかそういうのもあるかな.		
PtE397	1	もし私がここで弱音を吐いたら，だめでしょ.		
PtE398	10	まあね家族も待っていますしね.	家族の存在，家庭内役割があることがやる気を維持できる	
PtE399	10	うちは主人を早く無くしているんでね，私が全てをやってきたんです.		
PtE400	10	それを子どもたちも見ていますから，早く帰っておいでって言ってくれているんです.		
PtE401	10	家族のためっていう思いが大きいです.		
PtE402	10	家のことは気にしないで頑張ってって言ってくれているので.		
PtE403	10	誰も来てくれないと落ち込んだりしますよね.	友人の存在があることでやる気が維持できる	友人の存在

PtE404	10	友達も来てくれますし，本当にありがたいですよ．			
PtE405	10	環境って本当に大きいと思いますよ．			
PtE406	1	周りからっていうと，やっぱり家族でしょうね．	家族からの叱咤激励により向上する	家族との会話	支援者との関係性
PtE407	1	ここ入ったから一生懸命やって！って叱咤激励と言うか．			
PtE408	9	家族からも良くなったねとか，歩けるようになったねとか，孫がきても良くなったねって言われたりします	家族からの声かけにより向上する		
PtE409	9	そういう家族からの声かけっていうのもすごくありがたいですね．			
PtE410	9	すごく心配していましたし．			
PtE411	10	やっぱり少しでも動けるようになったっていうこと．	家族からの改善承認，改善の喜びにより向上する		
PtE412	10	家族が来たりなんだりして，良くなっている，進んでいるって喜んでくれる．			

PtE413	10	そういう家族とかの反応を見ているとやろうっていう気持ちになりますよね.	
PtE414	10	家族から来る度に良くなっているねって褒められるっていうのもありますよね.	家族からの改善承認の声かけにより向上する
PtE415	10	そういうのがあるから少しでも頑張って早く帰りたいなって思いますよね.	
PtE416	10	弟夫婦が毎週来てくれているので，その度に良くなっているのが分かるよって言ってくれるので，それが励みになりますよね.	
PtE417	10	あとは弟夫婦とか家族とかから良くなったねって言われるってこともありますしね.	家族からの改善承認の声かけにより向上する
PtE418	10	定期的に家族が来てくれるっていうのはありがたいですよね.	
PtE419	10	その度に座っていたり，経ったりするのが安定感が出てくると褒められたりもしますよね.	
PtE420	3	家族が来てくれて，がんばってねって励ましてくれたりした時っていうのは維持できますよね.	家族からの声かけにより向上する
PtE421	3	自分ひとりだと維持し続けることが難しいです.	

PtE422	2	みんな時間を割いて来てくれて，歩き方とか動きを見て良くなってるねとか言われると，もっと早く頑張って家に帰らないととかって思うよね．	家族からの改善承認によりモチベーションが上がる	
PtE423	2	次に来るときにはここまで良くなっていようとかって気持ちになるしね．		
PtE424	3	家族が来ないとかは影響しそうですよね．	家族の関わりが無いことで停滞する	家族の面会の要因
PtE425	2	孫達が来たときにもこれだけ良くなったんだぞっていうのを見せたいって思うよね．	孫がお見舞いに来てくれることがモチベーション	
PtE426	2	前の病院だと寝たきりで動きも全くとれない状態だったから，見てくれって感じになるよね．		
PtE427	2	孫達が来てくれたっていうのはすごく嬉しいよね．		
PtE428	2	来てくれるとまた早く家に帰って，家で会いたいなていうのがあるよね．		
PtE429	2	家族が来ることも早く帰りたいっていう気持ちになるよね．	家族面会により退院欲求が高まり，モチベーションが上がる	
PtE430	2	土日とか他の家族が来ているのを見ると羨ましいなって感じにもなるしね．		

PtE431	2	面会っていうのは，来てくれたんだっていう嬉しさがあるよね．				
PtE432	2	それがやる気にも繋がると思うんだよね．				
PtE433	6	やる気が下がるというかそういう時って言うのは，自宅からとか家族からのくだらない電話があった時ですかね．	自宅環境の負の変化により低下する			
PtE434	6	ましてや，自分の通りにならないことを言われたときとかは落ち込んだりしますよね．				
PtE435	6	息子とかからも言われますけど，親父ならこれぐらい言っても大丈夫だろうと思っているんですよね．				
PtE436	6	よく言えば叱咤激励ですけど，すごすぎるとね落ち込むことも有りますよね．				
PtE437	7	やる気がある時っていうのは，ここらへんの廊下を自分で歩くようにしたりしていますからね．	やる気が高い時に自主的に歩行訓練を行う	自主トレ時間	自主トレ	行動の変化
PtE438	7	疲れているときとかもあるけれど，体を良くしたいとか，やらなくちゃっていう気持ちのほうが大きいよね．				
PtE439	2	あまりゴロゴロしたりはしないし，テレビを見るってこともしないし．	やる気がある時は自主トレをする			

PtE440	2	そういうときには歩いて回ってみたり，まあそれも言ってみれば自主トレに入るよね.	
PtE441	2	あんまり時間があくっていうのは良くないよね.	やる気がある時は自主トレを行っている
PtE442	2	その間どうしようとかってなるし，退屈だなってなるし.	
PtE443	2	でもその時間を使って自主トレしたりするかな.	
PtE444	2	まあただ歩くっていうのもあれだなって思うときは，杖を浮かせて歩いてみたり，指導してもらったことを繰り返し考えてやってみたり，工夫しながら歩いたりするかな.	やる気がある時は自主トレも自分なりに工夫する
PtE445	3	リハビリが始める前から自主トレをしているというか.	高いときには訓練前でも自主トレをしている
PtE446	3	人からやりましょうっていうこともなく，自分からやりたいなって気持ちになります.	
PtE447	6	高い時って言うのは進んで，リハビリ室に出てきて療法士の先生に言われたことを練習しましたよね.	高いときには療法士が指示した練習をしている
PtE448	6	もうリハビリ以外で自主トレをすると足が棒になっちゃうんですけど，それはやる気が高いときじゃないとやらないよ.	

PtE449	7	私の場合は廊下を歩いたりしていましたかね.	やる気がある時は自主的に歩行訓練を行う	
PtE450	9	担当の先生方に教わったことを部屋でもできるかなって思って10回でもいいからやろうっていう気持ちになったりしますよね.	やる気があると療法士から指導された自主トレを部屋で行う	
PtE451	9	何回やっても出来ないときとかは私は逆にやろうっていう感じになるんですよね.		
PtE452	9	今は大部屋じゃないからお部屋で出来るじゃないですか？		
PtE453	9	なので，ちょっと広くなったので悔しくて色々やろうっていう感じでやっていたりしますよね.		
PtE454	1	自主トレはやったことがないね.	訓練に体力を残すため，自主トレはしない	体力温存のため自主トレの未実施
PtE455	1	自主トレまで体がもたない.		
PtE456	1	リハビリに出なくちゃいけないから，自主トレをしていると逆に疲れちゃってダメだから.		
PtE457	5	自主トレとかは，リハビリを頑張るためにしないことが多いですよ.	自主トレは訓練に影響するためしない	

PtE458	5	リハビリをするだけで着替えをしなくちゃいけないぐらい汗びっしょりなので，それ以上に自主トレをすると本来のリハビリに影響が出ますからね．	
PtE459	6	自主トレもそうだけど，やりたいって思っても体がついてこないってことも多いよね．	自主トレは体力が続かなくて出来ないことが多い
PtE460	6	やりたくても体力が続かないんだよ．	
PtE461	8	なにか変化したっていうのはないかな．	やる気の高低で行動は変化しない
PtE462	8	出来ることはやりたいけど，出来ないことはやりたくない．	
PtE463	8	モチベーションが低いとかっていうこともないし，そこまで行動としては変わってないと思う．	
PtE464	8	歩くとかっていうのもそれは自然と歩いているけどな．	
PtE465	8	それがモチベーションに左右されるっていうのはないよね．	
PtE466	10	昼間に自主トレとかはしないですね．	高くてもリハビリ訓練時間以外に自主トレは行わない

PtE467	10	リハビリの時間に外行ったりしているので.			
PtE468	10	看護師さんからあるきましょうって言われたときとかは歩きますけど.	やる気の高低で行動は変化しない		
PtE469	10	やる気の高低で行動がなにか変わるっていうことはあまりありませんよね.			
PtE470	5	高い時って言うのは，いろんなことに挑戦しようって思いますよね.	高いときには積極的に高難易度の課題に挑戦しようとする	訓練への能動的態度	能動的動作
PtE471	5	これはやりたくないなって感じたときはやる気がペースダウンしているなって思いますよね.			
PtE472	5	いろんなことっていうのはリハビリの内容でもそうだけど，自主トレでもやってみようっていう気持ちが自然と湧きます.			
PtE473	5	自分の能力よりも少し高い課題とか動きをやろうって感じる時って言うもの高いときかもしれませんよね.	高いときには高難易度のものに挑戦する		
PtE474	5	ペースダウンしているときっていうのは，自分が出来ることしかやらなくなる感じがしますよね.			
PtE475	7	疲れている時はやる気が無くなりますよね.	やる気が停滞している時は自室にこもりがちになる	自室滞在時間	

PtE476	7	外に出たりしたくないなとかっていう気持ちにもなりますし.		
PtE477	7	横になりたいなっていうこともあると思います.		
PtE478	3	朝に目が覚めて今日はすごく調子がいいなってときもあるんですよね.	高いときには病棟でも活動的になる	日常生活動作への能動性
PtE479	3	そういうときには朝から歩いてますよね.		
PtE480	6	あとは，空いている時間に好きなことをやったりしてますね.	高いときには趣味活動を行っている	
PtE481	6	他の趣味を持ってきているからね.		
PtE482	7	ある程度は制限されていますからね.	高い時は活動許容範囲内で出来るだけ活動的に行動する	
PtE483	7	自分でやりたいと思っていても，ここの階しか歩けないんですからね.		
PtE484	7	けど，自分が出来ることを考えて，じぶんが何が出来るかっていうのを考えてこの廊下を歩いたりしているんですよね.		

PtE485	8	体力を温存するっていうことも俺はないよな.	看護師との訓練も積極的に行う	
PtE486	8	看護師とも病棟でどんどん歩いているしね.		
PtE487	4	やる気が行動に影響するって言うことはあまりないですよね.	自発的行動への変化はなく，言われたとおりに行う	行動変化なし
PtE488	4	療法士の先生達がうまくやってくれるし，いい人たちだしね.		
PtE489	4	その人達とリハビリをしているときっていうのはやる気が下がるってことはないですよ.		
PtE490	4	言われたことをひたすらやっているって感じ.		
