# Supplementary material for: Motivation for Rehabilitation in Patients With Subacute Stroke: A Qualitative Study
Source: Front Rehabil Sci. 2021 Jun 7;2:664758. doi: 10.3389/fresc.2021.664758 (PMC9397769; doi:10.3389/fresc.2021.664758)
Supplement: Supplementary file 1 [file Data_Sheet_1.zip › data sheet/Coding of qualitative data in patients aged < 65(in Japanese).pdf]

S1 Appendix. Coding of qualitative data in patients aged < 65

ID	Subject Number	Record Unit	Code	Subcategory	Category	Core category
PtY1	1	何が出来るようになりたいっていうイメージが自分の中で具体的にあったほうがいいですよね.	将来への目標があることで向上する	活動目標からの要因	個人的達成目標	患者目標
PtY2	1	小さな目標でも何か持っている人は今リハビリでこういうことしようと考えていると思います.				
PtY3	4	目標設定って自分の目標ってすごく高く設定していたんですけど，ちょっとづつその手前のことが出来るようになっていった手応えとか，練習すればそれが出来るようになっていったとか，これはちょっと難しそうとか，自分の中でそういったのが有るんです.	自身の明確な目標があることで向上する			
PtY4	4	良いと思ったときにはどんどん練習をしますし，やる気にもなります.				
PtY5	1	体のことで悩んでいる方もいると思いますし，将来が見えないという人っていうのも悩んでいると思います.	将来の目標がないことで低下する			
PtY6	3	私事で恐縮なんですが，2 年前に妻を亡くしてるんです.	退院後の活動目標			
PtY7	3	僕が定年したら行きたいところがたくさんあったんです.				

PtY8	3	初めて行く所じゃなくて，付き合っていたときに行ったところをもう一度全部行ってみようねって言っていて，旅行先とかを決めていたんです.		
PtY9	3	でも家内とはできなくなってしまったので，一人ですけど家内の写真を持って家内とした約束を果たそうと思っていたんです.		
PtY10	3	ですけど，今の状況じゃ出来ないので，病気の前の状態に近づけて約束を果たそうと思っているんです.		
PtY11	3	そういう個人的な思いっていうのがあるんです.		
PtY12	8	現実問題，店をすぐやるっていうわけには行かないけど，まあ早く普通の社会復帰というか普通の生活をしてみたい.	病前生活への復帰目標	
PtY13	8	もちろん苦しいのはわかってるよ．よくよく考えると不便だし大変だっていうことはわかっているけど，でもやってみたいよね.		
PtY14	8	今までの自分の生活に 1 日 2 日戻ってみたい.		
PtY15	8	そこでさ，1 時的にはモチベーション下がるよ，こんなことも出来ないのかって．健康な体だと簡単に出来ていたことがこんなことも出来ないんだっていう気持ちもわかるけど，まあそれを試してみたいんだよね.		
PtY16	2	自分で体を良くしたいとか，体力をつけたいとかっていう目標ももちろんあります.	身体機能改善目標	機能能力改善目標

PtY17	2	歩行が不安で，杖を持って歩いちゃダメですかって聞いても，将来的には杖を使いますか？って聞かれちゃうと，２本の足で歩きたいですってなりますし，それに向けて頑張ろうって気持ちにもなりました．	歩行自立目標により向上する		
PtY18	2	高い目標を提示されてたときにも，元に戻ろうっていう強い気持ちが合ったので，モチベーションが下がるっていうことはなかったですね．			
PtY19	5	体が自由になるようにっていうことが一番大きいですよね．	病前身体機能開宴目標		
PtY20	5	元の体に近づけられればっていうのが大きいですね，			
PtY21	2	それはやっぱり本人の迷いだったりすると思うので，最終的には家に帰って主婦として仕事をするために頑張りますとか．	主婦役割再獲得	主婦役割	家庭内役割
PtY22	2	そのためにはこれをしないといけないとか小さな目標があると思いますし，それを一つ一つ提示することが大切だと思います．			
PtY23	2	私自身は父の介護をしなくちゃならないので，一日も早く退院したいっていう思いがモチベーションを高めましたね．	家族の介護役割再獲得が必要	家族支援役割	
PtY24	2	早く家に帰って父の介護をしないとっていう義務感みたいなものも有ると思います．			
PtY25	2	私自身は目標が有りましたけど，入院している方の中には目標を持てなくて，どうしようって悩んでいる方もたくさんいると思います．	家族の介護役割再獲得が必要		

PtY26	2	目標が有るかないかだと思いますよ.			
PtY27	2	元通りに戻って父の介護をしないといけないっていう目標がアリますしね.			
PtY28	7	やる気になるのは，ただ，自分の仕事に戻るっていう目標です.	復職意欲により向上	復職目標	社会的役割
PtY29	7	仕事に戻りたいって思ったときにはやる気が上がりますよね.			
PtY30	7	そのためには今リハビリをしないとって思いますし．戻りたいっていう目標が明確になっているっていうか.			
PtY31	1	なぜかって言うと仕事に復帰したいっていうのが有るので.	復職意欲により向上		
PtY32	1	そのためにはある程度元の状態は無理だとしてもそれに近い状態にならないと復職は出来ないって思うので．なのでリハビリをやりたいなっていう気持ちは強いですね.			
PtY33	1	それはずっとベースに有るモチベーションですよね.			
PtY34	1	社会復帰も動機を高ぶらせる要因に入っていると思います.	仕事と社会復帰目標により向上		

PtY35	1	早く仕事に戻りたいですしね.	
PtY36	9	最終的な復職っていうのはすっごく大きですね.	最終的な復職目標により維持
PtY37	9	積み重ねじゃないと出来ませんし, 魔法じゃないので急に良くなるとか, 家族が心配ないレベルで歩けるようになるとかなるわけじゃないので.	
PtY38	1	前の仕事に復帰したいとか, 目標が有るっていうことが大きいですね.	復職目標により維持
PtY39	1	目標を持ち続けるというか.	
PtY40	4	けど, 自分の中の目標である退院とかが近づいてくると, 職場でこういうふうに動くとか, こうするんだとか具体的にビジョンが出てきたりとか, どんどん具体的になっていくっていうのがモチベーションですね.	職場での必要動作イメージにより維持向上
PtY41	4	ここまでだとこれが足りないとか, ここがこうなると更に良くなるとか, ここにいて考えられる想定内のことはやっておきたいとかですね.	
PtY42	5	そして仕事に復帰したいっていうことですかね.	復職目標があることで向上する
PtY43	5	仕事したいとか体が良くなりたいとかっていう目標があるっていうのが大きいです.	

PtY44	5	今は休職中ですけどね，またそこに戻りたいなって.	
PtY45	7	やっぱり仕事と家に早く戻りたいっていう気持ちですね.	職務遂行可能な状態で復職したい
PtY46	7	仕事に早く戻っても迷惑がかかるようではダメじゃないですか？	
PtY47	7	ただ復職するだけなじゃくて，しっかりやりたいんです.	
PtY48	7	家族に対してとかは全然ないです．とにかく仕事です.	復職目標により向上する
PtY49	9	今は退院した後に動きですかね.	復職目標に向けての訓練が維持させる
PtY50	9	PT だったら杖使って歩けるようにとか，OT だったらなるべく出来ることを増やしたいとか.	
PtY51	9	最終的には僕も復職ですけど，今すぐにっていうのは無理だと思うし，段階を踏んで行かないとダメだと思うので，少なくともここで出来る限りのことはやっておこうっていう思いが有るのでそれをモチベーションにリハビリをしています.	
PtY52	10	療法士も医師もそうですし．アピールしないとみたいな．早く帰りたいっていう基本には早く復職したいっていうことがありましたよね.	早期復職目標により維持できる

PtY53	10	最初は，すぐに復職なんてっていう説明だったので，まずいなって思っていたんです．	早期復職可能性があることで向上する		
PtY54	10	でもちょっと出来るんだぞっていうところを頑張って見せてみると，出来るんですね？っていう反応になって，時期が早まるっていうことがあったんで．まだまだわからないぞって言う感じになったていうことも影響していますね．			
PtY55	1	ざっくりともとに戻るというか．体のことも含めて仕事もゴルフとかの趣味も元の状態に戻すっていうそれだけです．	趣味活動再開の目標により向上	趣味活動	
PtY56	2	あとは，自分の趣味でやっていることも継続して続けたいっていう思いもあります．	趣味活動再獲得目標により向上		
PtY57	2	個室なので趣味の一端をしてみたりしていますね．			
PtY58	1	あとは，飲みに行くのが好きなんで，ジョッキを持てないとダメですしね．	友人との交流再開の目標により向上	友人との交流	
PtY59	1	温泉も好きなので，また温泉には行きたいなって思いますし．			
PtY60	1	ざっくりともとに戻るというか．体のことも含めて仕事も趣味も元の状態に戻すっていうそれだけです．			
PtY61	10	時期によっても若干違うように感じますけど，入院当初の予定期間からもしかしたら長引いちゃう可能性もあるわけですよね．	早期退院への思い		自宅退院目標

PtY62	10	とにかく早く退院するためにがんばらなくちゃっていうのが有りましたね.	
PtY63	10	そのためにはある程度認めてもらわないといけないっていうことがあるじゃないですか.	
PtY64	10	最初は装具を作るとかもあったんですけど, ないにこしたことがないじゃないですか. じゃあがんばろうって.	早期退院への思いで維持できる
PtY65	10	もう最初はがんばろうしかなかったですよね. 切迫感というかそういうのが大きかったと思います.	
PtY66	10	自分の中でのタイムリミット的なタイムスケジュールがあったんで.	
PtY67	10	それをモチベーションにがんばっていたかなって.	
PtY68	6	早く家に帰って普通の生活に戻りたいって思うし, それのためにはやることやらないとかっていう気持ちが大きい.	早期退院目標があることで維持できる
PtY69	6	仕事もあるけど, それは普通の生活を続けていくうちに仕事に戻れるような自信がついてくるかなって思う.	自宅退院目標があることで維持できる
PtY70	6	まずは普通の生活に戻るってことが先だよね.	

PtY71	6	普通の生活をしていく中で，体ももう少し動くようになって，喋るのもスムーズになって来るんじゃないかなって思っているんだよね．		
PtY72	6	そうしたら仕事についても考えられるようになるんだと思う．		
PtY73	6	仕事では普通の会話っていうのが重要なんだよね．書いてある文章を読むんじゃなくて，	自宅退院目標があることで維持できる	
PtY74	6	思ったことをパって言えるようになるっていうのが俺の仕事では大切なんだよ．		
PtY75	6	紙をただ読むだけじゃしょうがないから，心から出るような会話が必要だし，そういう会話をしたいんだよね．		
PtY76	6	そのためには，まずは普通の生活に戻るってことが大切なんだよ．長期的には仕事も考えているけどね．		
PtY77	8	やっぱり早く家に帰りたい．	早期自宅退院希望で維持できる	
PtY78	8	帰って何したいっていうと．何したいんだろうな...		
PtY79	3	自分の予定では，今頃杖無しで歩けて，退院までのまとめをしているはずなんですよね．	予測期間との差により低下	現状と目標との乖離

PtY80	3	生活での工夫とかを聞いて準備するはずが，痛みのせいでまるまる一ヶ月伸びっちゃっているんです.	目標期間と現状に差が出ている			
PtY81	3	目標とかビジョンが崩れてしまっているということがモチベーションを下げていますね.				
PtY82	1	でも，基本的には下がることはないです.	改善実感による影響	機能能力改善	成功体験	成功失敗体験の要因
PtY83	1	日々良くなっているというか，伸びているからですかね.				
PtY84	2	良くなってきたっていう感覚があるっていうのがまたモチベーションを上げる要因になっていると思います.	機能能力改善実感により維持			
PtY85	2	モチベーションを上げるっていうのは自分自身では出来ないことも多いので，療法士の方たちの指示通りに動いて，アドバイスを頂いて，出来なかたことが出来ていくっていうことでモチベーションを上げることが出来ましたね.				
PtY86	3	訓練をやることによって，1 週間後にはできなかったことが出来るとか，動かなかったところが動くとか.	訓練効果実感により向上			
PtY87	3	そういったことが連続してあると非常にモチベーションが高くなりますよね.				
PtY88	5	昨日までできなかたことが出来るようになった時.	訓練効果実感により維持			

PtY89	5	足が動くようになったとか．起き上がるのがうまくなったとか．	
PtY90	5	ここにいる限りだと，社会復帰はまだ見えてきていないので，身近な動作が動くようになったとか方がやる気が出ます．	
PtY91	6	高くなるのは，少しづつ出来るようになってきた時かな．	認知機能改善実感により向上
PtY92	6	体がっていうこともそうだけど，頭がっていうこともあるかな．	
PtY93	6	前にできなかったことが出来るようになってくると，よし！ってやる気が上がるよね．	動作能力向上によりやる気も向上
PtY94	6	自分自身できるっていう自信がついてくるのはすごく良かったよね．	
PtY95	6	療法士がやってくれるテストの点数よりも，実際の動作とか行動とかで成果が出たときのほうがやる気が上がりましたね．	能力改善を実感するとやる気も向上する
PtY96	6	でも，テストの点数がこれだけ良くなっていましたよって言われても嬉しいけどね．簡単に喜ぶんだよな俺．	
PtY97	8	例えば，日々リハビリをしていて，箸の練習とかもそうだけど動くじゃんって思うじゃん？	能力改善実感により維持できる

PtY98	8	昨日まで全然できなかったのに，今日やったら出来るようになってるんだねとか，動くようになってるんだねっていうこういう発見が大事だと思うんだよ．	
PtY99	8	だからそういう発見が毎日有るからさ楽しいんだよ．自分としても驚きが有るんだよね．	
PtY100	8	日々良くなっている感覚が有るっていうのが大切だよね．	
PtY101	8	筋トレとかは苦しいよ，でも次の日どうなっているかって言うと，杖無しで歩いていて，前よりは良くなっているってなれば，その前の日の効果がどんどんついて来たんだなって思えば前向きになるじゃん？	
PtY102	8	だから俺は後ろ向きになるっていう人がわからないんだよ．	
PtY103	8	俺も小さい変化とかはわからないんだけど，第三者からみたら違うんだろうなって思うよ．良くなっているって思うよ．	
PtY104	1	やっぱり手ですよね．	機能能力改善実感により維持
PtY105	1	はじめは全然動かなかったのが，それがちょっとづつ動くようになってきて，少しでもいいから使ってみなって言ってくれて，フォークとか使えるようになって，使えるんだって思いました．	
PtY106	1	努力すれば動くようになるんだなって言うことが分かったことで，もっと使うようにしようって思いましたね．	

PtY107	1	目に見えて動きが良くなっているので，それをもっと良くするためには，自主トレも必要だなって思います．	機能改善実感により維持
PtY108	1	課題とか宿題ももらっているんで．自主トレの内容が多いなって思いながらも頑張ってやっています．	
PtY109	8	こんな体だと多少なりとも誰かの手を借りないといけないっていうのは分かるけど，自分のやれるところまでやってみたい．	効果実感がやる気を維持させている
PtY110	8	最終的には自分自身に納得したいんじゃないかな．	
PtY111	8	自分が精一杯やった結果がこれだぞっていう．	
PtY112	8	だってもしかしたら今よりもっと動けるかもしれないじゃん？そうだったら頑張ろうかなって思うじゃん．	
PtY113	8	ここにいるよりも実生活，実社会に出るほうがよっぽどのリハビリだぜ．	
PtY114	8	だれも手伝ってくれないんだから，自分でやるしかないんだから．	
PtY115	8	まあ確かに苦しいかもしれないけど，もしかしたら今以上に体が動くんだとしたらやりたいよね．	

PtY116	8	若いしさ，こっから先が長いんだから．	
PtY117	8	倒れたときから，なんでかなって思ったけど，でもしょうがないよね．	
PtY118	8	治るのかって言われても，すぐには治らないしさ，受け入れるしかないしさ．受け入れてリハビリしてなんとかここまでなってるけどさ．	
PtY119	8	２ヶ月前を考えてさ，足も口も動かなかったのが，商店街まで歩いて行けてるんだからさ．	
PtY120	3	成果が目に見えて分かって，その次の目標を提示されて，それに向かって厳しい訓練をするとかも重要ですね．	成果実感により維持
PtY121	4	自分なりに練習をして，療法士の方から指導を頂いて，素人なので何もわからないのでついて行ってみると，こないだは全く出来ていなかったのが，今日できたとか．	能力向上実感により維持
PtY122	4	とういうことがあるととりあえず前に進むことを考えるようになります．	
PtY123	5	なんというか，その日その日を一生懸命にやって，それが成果が出ているってことかな．	訓練中の効果実感がやる気を維持させる
PtY124	5	腹筋運動一つでも，いつもは５回やっっているだけだけど，今日は１回ひねりを入れて出来たなとか．	

PtY125	5	日々変化しているってことが大切ですね.	
PtY126	7	自主トレとかやってきてどんどん力がついてきて良くなって行ったときです.	改善維持がやる気を保たせている
PtY127	7	良くなっていることを実感しているっていうのかな.	
PtY128	7	良くなり続けているっていうのが大きいですね.	
PtY129	1	体が良くなり続けているっていうのも一つの要因かもしれないです.	身体機能向上継続が維持につながる
PtY130	7	今はある程度は出来るようになったかなって思います.	復職に耐えうる機能改善実感により維持
PtY131	7	職人なんで，力仕事じゃなくて私にしか出来ないこととかが有るじゃないですか.	
PtY132	7	それは少しづつ出来るようになっている感じまします.	
PtY133	1	とにかく健康な状態というか，五体満足と言うか，全部動きが完璧な状態で退院に向けてどんどん進んでいますしね.	身体機能改善により維持

PtY134	8	やる気が下がるってことはまったくないね．	機能能力改善維持によりやる気維持		
PtY135	8	例えば，作業療法士におはじきを拾いましょうとかってなるじゃん？			
PtY136	8	でも全くできなかったとして，でも二日目三日目になると出来るようになってきたってなると，時間が立つと体としては出来るようになっているんだなって感じるわけ．			
PtY137	8	毎日毎日良くなり続けているっていうことじゃないかな．			
PtY138	8	毎日良くなっている箇所が見つけられているかっていうと微妙だよ．でも昨日よりは確実にいいかなって思っているからさ．			
PtY139	8	本当に回復する能力があるし，昨日より悪くなっていることはないかって．			
PtY140	1	先生との訓練のときにパフォーマンスが出せなくてまずいなあって思ってやる気が下がっちゃったときも有りました．	前日との動作の質の差	実動作と期待した動作の差	失敗体験
PtY141	4	できなかったときとかも，昨日より全然だめじゃんっていうのは自分でも分かりますし，もうだめかなっていう気持ちにもなってしまいますね．	前日との能力差を感じることで低下する		
PtY142	7	自主トレも成果が停滞しているとあまりやる気にならなかったりするんですけど，やらない日っていうのはなかったですね．	改善停滞の影響	改善停滞の影響	

PtY143	6	やる気が下がるときっていうのは，何回やっても間違えちゃうときっていうのはやる気が下がっちゃうよね.	失敗体験の繰り返し	失敗体験の繰り返し		
PtY144	6	しかもそれを言われるとすごくやる気が無くなっちゃうんだよね.				
PtY145	10	下がるときは，少し疲れたりすると下がりますよね.	身体的疲労により低下する	身体的疲労	身体状況	心身状況による要因
PtY146	10	自主トレも確かに最初はしていたんですけど，なんか途中から疲れてやらなくなってきなって思いますね.				
PtY147	5	体的に言うと，疲れを残さないってことが重要です.	疲労の蓄積により低下する			
PtY148	5	翌日に疲れを残らない程度に訓練をやるとか.				
PtY149	10	例えば，周りからの励ましとか刺激もなくて，体調もイマイチで，なんか進んでるのかもよくわからないような状態だともしかしたらモチベーションが下がったままっていうことも有りえるかも知れないですよね.	体調悪化により低下	身体的虚弱		
PtY150	1	体調が悪いときとかっていうのはやる気が下がるのもそうですけど，療法士に悪いなって思いました.	体調の悪化により低下する			
PtY151	3	本来とは違う症状が出てきて，現状維持ならまだいいんですけど，下降してきているって感じたときはモチベーションが下がりましたね.	疼痛の出現により低下する	疼痛		

PtY152	3	本来痛くないところがどんどんどんどん痛くなってきて，通常の訓練すら出来ないっていう状態が続くと，モチベーションがも低い状況が続きますね．	
PtY153	3	しかも，痛みって自分じゃどうしようも出来ないっていうものですし，その状態にハマるとモチベーションががーんって下がりますね．	
PtY154	8	痛みじゃないかな．	疼痛の出現により訓練の意欲が低下する
PtY155	8	体が痛いとか．動かない体を動かすから痛いと思うよ．	
PtY156	8	だけどそれがずっと続くわけじゃないしさ，痛いんだったら違う方法で訓練してみるかなとか思うもん．	
PtY157	8	自分で解決策を思いつくというかやってみるっていうのがあるかないかもあるかな．	
PtY158	8	自分ももっともっといいたかったらもしかしたらモチベーションが下がっているかもしれないね．	
PtY159	3	低くなる要因としては，リハビリを頑張りすぎちゃって，本来の症状とは違うところに悪いところが出てきたときですね．	疼痛の出現により低下する
PtY160	3	本来の症状とは違う症状なんですけど，それを治そうとしている療法士の先生がいて．本当に頭が下がるんです．	

PtY161	3	でもまた違う症状が出てきてしまったりするんですよね.		
PtY162	3	痛みも出てくるし，症状も出てくるし，原因がわからないままっていうのが長く続くと，活動量も減りますし，リハビリの内容もおとなしくなってきてしまうということがありますね.		
PtY163	3	一番大きな原因としては痛みが出現して，それによって本来リハビリをして良くなるはずなのに，痛みによって時間が取られてしまっているって思うとモチベーションが下がってしまったりもしましたね.	疼痛の出現により訓練が出来ないと低下する	
PtY164	2	やりたくないなって，やる気が落ちちゃうのは筋肉痛の時.	筋肉痛の出現により低下する	
PtY165	2	痛くてどうしようもない時には，自分でストレッチとかやっていたんですけど，駄目かなって思うことは有りました.		
PtY166	2	体調がすぐれないときって言うのはモチベーションが下がりますよね.		
PtY167	1	やる気が無くなる時っていうのは，ガツガツやりすぎちゃって筋肉痛になったりしてどうしようかなって思うことも有りましたね.	筋肉痛により低下する	
PtY168	10	しびれ感とかも有りましたけど，そこはそう簡単に治らないんだろうなって思っていますし，まあ別にあんまり考えてもしょうがないかなって思っています.	痺れの出現により低下する	痺れ
PtY169	9	体の状態が変わったときですかね.	痺れの出現により低下する	

PtY170	9	具合が悪くなるとか．今まで感じてない痺れを感じ始めたとか．				
PtY171	9	それでも，良い方に変わっているよって言われれば抜け出すことが出来ますし，悪い方に捉えればモチベーションは下がりっぱなしになると思います．				
PtY172	9	疲れも有ると思いますし，そういうのが続くとか，悪い方に変わるとかがあればモチベーションは上がらないと思います．				
PtY173	8	だってさ，こういうふうな体になっちゃったのはしょうがないって認めているわけだからさ，	自身の障害の受け入れと切り替えが必要			障害のリジリエンス
PtY174	8	だったらこっからこの体で元の生活に戻していくしかないんだからさ．				
PtY175	8	たまには俺も寝る前に考えるよ．これからの生活はどうするのかとか．				
PtY176	8	前の状態には戻れないなとか．外に出るとおばあちゃん連中にさ追い越されてさ，こんな体になっちゃったのかって思うこともあるけど，このまま止まるわけにもいかないしさ．これからも先行きていかなくちゃならないんだしさ．				
PtY177	6	結構道を間違えていたんですけど，また間違えたよ，また間違えたよって言われるんだけどさ，その時はやる気が無くなっちゃうんだよね．	訓練中の負のフィードバックで低下	療法士からのフィードバック要因	療法士との関わり方の要因	専門職からの要因
PtY178	6	出来ないことを言われるのは凹むだけだったな．				

PtY179	6	もうなにやってもダメなんだっていう感じで．	
PtY180	9	療法士とかに否定的なことをいわれちゃうとモチベーションは下がりますよね．	療法士の負のフィードバックで低下する
PtY181	9	患者は不安は絶対に抱えていると思うので，それに対して解消するっていうことは無理でも同意というか，ちょっとでも乗ってきてくれていればモチベーションは上がると思います．	
PtY182	9	ここにいる人っていうのは最初はすぐに歩けるようになると思っているんですよね．	療法士の負のフィードバックで低下する
PtY183	9	そういう気持ちを持ってきているので．そこを否定的な感じで捉えられるとモチベーションが保たないですよ．	
PtY184	6	周りと比較して，自分が出来ていないいって気付くとか，療法士に言われるとかっていうのは落ち込むよね．	療法士の負のフィードバックで低下する
PtY185	6	このままじゃどこ行っても迷子になるよって言われたときは落ち込んだな．	
PtY186	9	否定的なことばっかり言われると気分的に乗ってこないなっていうのがありますね．	専門職からの予後提示により低下
PtY187	9	否定的なっていうのは，体についても動きについてもそうだけど，今後見込みがない，可能性がないっていうような言われ方をしちゃうと下がりますよね．	

PtY188	9	言いたいことはわかりますよ，こういうこと言いたいんだなって，	
PtY189	9	でも言い方をうまく言わないとやっぱり落ち込んじゃうなって感じはありますよ．	
PtY190	9	僕らは希望がなくなるとリハビリをしようっていうモチベーションも下がると思うんです．	
PtY191	4	本当のことも言ってもらわないとダメだと思うんです．	適切なフィードバックにより向上する
PtY192	4	すごい大事に大事にされると，ちょっとどうしたらいいのか分からなくなります．	
PtY193	4	結構はっきりと良いところとダメなところをはっきりと言っていただいて，じゃあどうすればいいかっていうのを言ってくださる方が良いですね．	
PtY194	4	良いことばっかり言われるのも困ります．	
PtY195	1	でもやっぱり褒められることが嬉しいって思うことは多いですよ．	専門職からの褒めによるやる気の向上
PtY196	1	知っている先生に声をかけてもらえるっていうのも嬉しいですよ．	

PtY197	8	療法士からの励ましの効果もあると思うよ．前向きになれるきっかけだからね．ここに入院して後ろ向きになったことはないよ俺は．	療法士からの褒めにより向上する
PtY198	10	でも実際にずっと維持するのは難しくて，家族とか見舞いに来てくれる人とかの励ましとか，先生，看護師，療法士の皆さんの励ましもですかね．そういったものがあるとモチベーションが高くなりますよね．	療法士の褒めにより改善を実感しやる気も向上する
PtY199	10	良くなったねって励ましてモチベーションが上がる前提としては，自分自身良くなっている感覚があるというか，	
PtY200	10	まあ後退はしていないなっては感じていますよね．徐々には少しは良くなっているのかなって思いますね．	
PtY201	10	本来の目標からするとまだまだですけど，それでも前よりは良いのかなっていう認識ですよね．	改善承認のフィードバックにより向上
PtY202	10	でもそれを療法士とか，医師とか看護師の方に良くなっているっていうことを示してもらうことはとても重要だと思います．	
PtY203	1	あとは，自分自身が何が出来るのかを療法士に報告をして褒められた時っていうのはやる気が高まりました．	療法士からの褒めの影響
PtY204	10	自分としてもできなかったことが出来るようになったとか有るんですけど，目指す目標からするとまだまだギャップが有るわけじゃないですか．	改善承認のフィードバックにより向上
PtY205	10	今自分はその目標に少しづつでも近づいているのかがわからないんですよね．患者は素人なので．	

PtY206	1	やる気はずっと高いですよ．先生との相性とかも有ると思います．	療法士との相性により向上	療法士への信頼感
PtY207	1	自主トレをしたいので，いろいろと課題を貰ったり出来たほうがいいですね．		
PtY208	1	ガツガツしている方が私としてはあっていると思うんです．		
PtY209	1	可愛い女の子が担当してくれるっていうのもありますね．	女性職員の存在により維持できる	
PtY210	1	病院には若い人が多いじゃないですか．それもいい環境だなって思いますよ．		
PtY211	4	訓練の最初とか練習中は当然療法士とか医師とかのためっていうのも有ります．	療法士の患者への寄り添いにより維持	
PtY212	4	一人ぼっちじゃないっていうのは，医師とか患者とかも大事ですけど，療法士の方が一番身近ですし，一番状況を知っていてくれていますし．		
PtY213	3	療法士の担当がよくやってくれていて，僕にすごく合っている先生でしたし，半分スパルタですし，やってくれることもハードなことも合ったんです．	療法士との相性により向上する	
PtY214	3	でも，それが必ず自分の快方につながっていたんです．		

PtY215	3	自分のためになっていったことがやっぱり大きかったですね.		
PtY216	2	何が何でもやらせるということではなくて，相手の信条を理解することが大切です.	患者とのコミュニケーションが重要	
PtY217	2	相手の意思を引き出すようにするとその人のモチベーションにつながるのかなって感じますね.		
PtY218	3	リハビリの先生が非常に熱心に僕が投げかける問題に対して答えてくれるときにモチベーションが上がります.	療法士の熱意があることで維持	療法士の患者に対する態度
PtY219	1	先生とか周りからの褒められるっていうのは，本当に思っているのかなっていうのも有りますしね.	褒めは影響しない	褒めの影響はない
PtY220	1	確かに言ってくれることが有るんですけど，本当かなって思っちゃうこともあります.		
PtY221	3	先生からとか周りからの賞賛とか褒めっていうのは本当わからないですし，全くモチベーションに影響しませんでした.	褒めは影響しない	
PtY222	4	だからといって，明らかにかばったような言い方とかも違うと思うんです.	過度な賞賛は影響はしない	
PtY223	4	本人たちも病気のことについて調べたりもしますし，今はネット社会なのである程度情報として知識を持つことが有ると思うのです.		

PtY224	8	看護師とのやり取りとかは影響するかな.	看護師からの声かけの影響	看護師からのフィードバック要因	看護師との関わり方の要因
PtY225	8	担当看護師がいて，オレンジカード（一人外出許可書）をもらったときに俺自身ここまで来るとは思ってなかったよって言ったら，立ち上がり訓練の時から見ていて村山さんは絶対にここまでくると思っていたよって言われたらそこまで見ててくれたんだって思って嬉しかったな.			
PtY226	10	そういうときに，こんなに出来るようになったんだとか, 良くなったねとか看護師さんとかに承認してもらうことってすごく大事だなって感じます.	看護師からの改善承認により向上する	看護師への信頼感	
PtY227	1	家族だけじゃなくて，看護師とか周りからの声がけとか承認されるってことも大切だって思います.	看護師からの改善承認により向上する		
PtY228	4	下がるときっていうのは，主治医にもうだめだよって言い放たれてしまう時.	医師からの予後提示により低下する		医師との関わり方の要因
PtY229	4	例えば可能性としてはゼロじゃないわけで，少しでも 1％でも可能性が残っているのであれば，ダメだよっていう表現じゃなくて，表現を工夫するほうがいいと思うんです.			
PtY230	1	特に親指の動きがあまり出なかったんですけど，新しい装具とか出してもらえてまたやる気がでました.	療法士の訓練方法提示	訓練バリエーションの豊富さ	訓練方法
PtY231	3	その答えが合っているかどうかわからないけれども．本当にいろいろと考えて，A 策がダメなら B 策を．B 策がダメなら C 策をっていうように，自分が調べてきたことの範囲の中でいろいろと提案してくれるとき.	多くの訓練方法の提案により向上する		

PtY232	3	代行で入ってくれた療法士の方たちもまた違う視点からの解決策を教えてくれる.	代行担当者からの別視点
PtY233	3	どんどん自分に合ったリハビリテーションをずっと継続して与えてくれているときは，こっちもやらないとっていう気持ちになりますよね.	多くの訓練方法の提案により向上する
PtY234	8	高くなるっていうか，日々ちょっと変化があるじゃない？	代行担当者による気付きにより向上する
PtY235	8	担当はいるんだけど代行者で違うことを教えてくれるというか，気づかせてくれるっていうか，それがすごく良いんだよね.	
PtY236	8	もちろん担当は担当ですごく良いんだけど，また別の考えでここをこうしたらどう？とかっていうことを気づかせてくれるっていうのがすごく良い.	
PtY237	8	それを気づかせてくれるって言うことで気持ちが上がるきっかけになる.	
PtY238	1	療法士によって新しい課題が出されるっていうもの有るじゃないですか.	代行担当者による気付きにより向上する
PtY239	1	療法士の特性というか，目標は一緒でもやり方が違うっていうのか. 最近だと輪ゴムを使用しての訓練方法とか教えてもらったんですよ.	
PtY240	1	その人ごとでやっぱり持っていう訓練内容が違いますよね.	

PtY241	4	代行でいらっしゃる療法士の方に対しては，なかには専属の人じゃないと嫌だって思う方もいるかもしれませんが，私の場合は体力をつけていこうっていう人もいたり，理屈で攻める人もいたり気づきを与えてくれる人だったり，女性の細やかさであったりっていうのはよかったかなって．	担当と代行者の訓練の進め方の違いにより気付きを促される	
PtY242	4	いろんな療法士がいてメインの療法士がいるっていうこのシステムは私は非常にありがたいと思っています．		
PtY243	9	与えられているだけなじゃなくて，やっている内容を理解して自分自身納得して訓練を行っているとリハビリへのモチベーションが上がると思う．	患者療法士との間での訓練目的共有	療法士と患者の訓練目標共有
PtY244	9	やることに対する意味とかをしっかりと説明してもらわないと，フィットしないと思うんです．	訓練目的の理解が維持には重要	
PtY245	9	分からなくてやっていてもおもしろくもなんともないと思う．		
PtY246	9	しかも，その目的が自分が足りていないいって感じて納得している物であればなおさら．		
PtY247	9	目的がないとモチベーションはもたない．		
PtY248	2	リハビリっていうのはただの筋トレじゃなくて，先生と一緒に目標をたてた実際の生活に基づいた訓練を一緒に組み立てて行くことが大切だと思います．	療法士との目標共有が重要	
PtY249	4	言葉を受ける人の能力にもよると思うんですけど，言っていただいたうえで，ここは頑張っていきましょうとかの表現があったほうが，信頼関係が作られます．	訓練ごとの目標提示により向上する	

PtY250	4	任せたいっていう気持ちも出来ますし，自分自身がんばってみようと思います.		
PtY251	4	今の訓練の目的と，自分が目指している目標があっていないときっていうのもモチベーションは下がりますよね.	患者療法士間での訓練目的の相違により低下する	
PtY252	4	僕はたくさん喋るので，先生の意見を聞くし，目的がどうなのかっていうのも聞きます.		
PtY253	9	でもなかには聞けない患者さんもいると思いますし，聞いても合わないなって感じる患者さんがいるかもしれないです.		
PtY254	2	目標があって，それに向けて一つ一つクリアしていくことが大切だと思いますね.	短期目標の決定方法	訓練内容の適切さ
PtY255	2	クリア出来ていなくて次の課題を与えられてしまうととてもつらいと思うんです.		
PtY256	2	一つづつクリアしていく中で，徐々に訓練の内容を付加していってくれて，その過程を療法士の方たちが導いてくれるっていうことが私たちのモチベーションアップに繋がると思います.		
PtY257	2	最初来たときにも階段で 4 階まで上がるっていうのはすごく辛かったんです.		
PtY258	2	でも，辛いからやめるっていうのではなくて，1 歩づつでもすこしずつやろうって思うことでたどり着けたって言うことも有りました.		

PtY259	4	大きな目標があってもそこに向かってなにをしたら良いのかっていうのは，本当にわからないんです.	目標設定と提示により向上する
PtY260	4	そこに向かってカリキュラムを組んでいただいて，自分自身がどうなりたいのかとか聞いてもらって，できそうにないこともあったんですけどね.	
PtY261	4	それを真剣に聞いてくれて，なんとかそれに向かって出来るようにって用意してくれると，療法士のためにも頑張らなくちゃっていう気持ちにさせられますね.	
PtY262	4	ダメでもともとっていう気持ちもあります．だけど私はこうなりたいっていう気持ちもあるので，それを引っ張っていってもらえるとついていこうっていう気持ちになりますね.	
PtY263	2	達成可能な目標をコンスタントに提示されること.	短期目標の定期的提示により向上する
PtY264	2	最終的な目標があるから，それに向けてモチベーションを維持しながら近づいて行くのは大切ですよね.	長期目標に向けた短期目標設定が重要
PtY265	2	最終的に自分はどうしたいとか，どうなりたいとかそういう目標と道順がないと難しいと思う.	
PtY266	2	それは，入院しているなかで療法士の方に引き出して頂くことが大切.	
PtY267	9	ある程度，リハビリテーションの見通しがないとついていけない. 短期的なゴールがないとモチベーションが維持できない.	長期目標に向けて目標提示で向上する

PtY268	9	最終的には大きな目標っていうか復職とかになるかなって思います.	
PtY269	9	けど，時間的な問題も有るので，ここでやるのはここまでですよ，絶対頑張りましょうねお互いにっていう感じだとイケルと思います.	
PtY270	9	実現可能な目標を適宜与えてくれるというか.	
PtY271	3	そういった大きな目標があって，それに向けた小さな目標として体を良くするっていう小さな目標がある，	短期目標遂行が維持には必要
PtY272	3	それを糧に日々のリハビリテーションを行っていますね.	
PtY273	1	先生によっては時期を決めて，ここまでには出来るようになりましょうとかって決めてくれることが有るじゃないですか.	訓練難易度調整が重要
PtY274	1	そうすると何とかしてそれまでに頑張らないとって思うんですよね.	
PtY275	1	目標設定も高くてもダメだし，低すぎてもダメだし，適度な目標設定をしてもらえることが大切だと思います.	
PtY276	4	本人としては，何をしていてもこのためにやっているとかは意外と分かっていないんです.	新規訓練方法の場合は難易度調整が重要

PtY277	4	ただ指導されたことをやっているだけで，難しさとかは言われないとわからないと思うんです．	
PtY278	4	なので，出来ないことが続いているときとかは，昨日とかその前とかに戻って，本人が得意そうなことをやるとかが良いと思います．	
PtY279	4	出来ないときとか, 更に悪くなっているって思うときも有るんですが, そういうときに出来ることをさせてもらえるとかも大切だと思います．	困難な動作訓練では難易度調整が重要
PtY280	4	出来ないところを攻めていくっていうことはとても大切なことだと思うんです．	
PtY281	4	けど, その人の波というか, バイオリズムを見て落ちているときにはその人の得意なことをやってあげるとかが患者さんのやる気をゼロにしない，マイナスにしないっていうことに重要だと思います．	
PtY282	3	背景にあるのは，リハビリテーションっていうのは患者さん自身はわからないと思っているんですよ．	訓練難易度調整の適切さにより向上する
PtY283	3	どこをどうやってどうしたらここの部分が良くなるのかとか．でも療法士の方はそういうことを全てわかっていると思うんですよ．	
PtY284	3	まあ，それでも解決できないことがあると思うんですけど．色んな方法を実践してくれて, 出来そうな内容のものからやってくれて, それの積み重ねで維持出来るものがモチベーションだと思います．	
PtY285	4	すごく体調の良いときもあるし, 病人って体調の波が激しくなっているので, ちょっと悪いときには無駄に集中して頑張らせたりすると，逆にどんどんできなくなっていってしまうこともあると思います．	訓練難易度の柔軟な調整により向上する

PtY286	3	やる気があがらないっていう状況から抜け出すっていうきっかけを作ってくれたのも療法士の先生では有りますね.	解決策提示が維持には重要
PtY287	3	根本できな解決ではないんですけど, いろいろと考えてくれて, 痛みが出ない動作を見つけて，それを今は頑張ってやっています.	
PtY288	4	目標があってそれに向かって今何が足りていないのかっていうのが明確に分かってくるとモチベーションに繋がると思います.	問題点を患者自身が理解することが重要
PtY289	6	できるようになるっていうのは, その日に間違えたことを夜に振り返って「なんで間違えたのかな.」っていうのを考えて，次の日のリハビリのときに，もう一度同じ道を行ってくれって頼んで, 質問攻めにしていろいろ聞いて頭に入れて, 自分の中で間違えたところが分かってくると出来るようになってやる気も上がってくるね.	問題点を患者自身が理解することが重要
PtY290	6	なんで失敗したかを自分で振り返って, 考えてどうすればいいかていうのを考えて, 自分でやっていこうって思っているときっていうのはモチベーションが高いかな.	
PtY291	6	なんとか工夫して失敗しないようにやっていこうとか思っているときっていうか.	
PtY292	6	そういう時っていうのは, 昨日と同じ道を行ってくれとか頼んだり, 間違った箇所をなんで間違ったか聞いてみたり質問攻めしたりしていた時には，なんとかやってやろうってやる気が高い状態だったなって感じるな.	
PtY293	6	自分でどうしたら良いのかっていうのがわからない状態が続いているときっていうのはやる気が上がらなかったな.	問題解決策が不明確さにより低下する

PtY294	6	ダメなところはわかっているけど，どうしたら良いのかわからないというか.	
PtY295	6	解決策がわからないときっていうのは何をしていいかわからないからやる気も沸かないよね.	
PtY296	7	歩き方がどうみてもおかしいなって思って，見かけが悪いなってわかるんだけど，どうしたら良いかわからない時かな.	問題点解決策が不明確な時期は停滞する
PtY297	7	療法士が一生懸命にやってくれるんだけど，言われたことがわからないときにはどうしたら良いんだろう，って思っていました.	
PtY298	7	でも今は少し良くなったのと，分からなければ聞いてます. いろんな人からいろいろと聞いてます.	
PtY299	7	やる気が落ちるのは，歩き方がわからなくなっちゃって，どうやったらいいのかわからない時ですね.	問題の解決策がわからない時は停滞する
PtY300	7	今まで良くなってきて成果も出てるなって分かっていたんですけど，それ以上ってなるとどうしたらいいのか，途中でよくわからなくなっちゃって. そのときにはいろいろ療法士の人に聞きました. なんか急ぎすぎたのかなっていう感じもありました.	
PtY301	3	自分が出来たことというのが，療法士の訓練とか提案とかだった場合はまたモチベーションが上がるし継続していくと思います.	療法士の課題提案方法により維持できる
PtY302	3	そうすることで，その先生についていこうとか一緒にやっていこうっていう気持ちが高まると思います.	

PtY303	4	周りからの影響とかですと，単純にただ見てみて前よりも元気になっている方とか．病状が違っていても進行が違うっていう方でも元気な姿をみると励みになって，自分自身も頑張ろうっていう気持ちになります．	他患の頑張りを見ることからの影響	他患の努力を見ることの要因	他患観察からの要因	患者関係からの要因
PtY304	4	周りに引き上げてもらっているというか．				
PtY305	4	比べてとか，競争とかは，密かには合ったかもしれませんね．	他患者の退院決定が自身の励みになる			
PtY306	4	良くなったらおめでとうって思うし，自分も負けないように頑張ろうとかも思います．				
PtY307	4	頑張って退院が決まったっていうのを聞くと，自分も頑張って退院しようとかって思うこともあります．				
PtY308	4	そういう気持ちっていうのは少なからず皆さんお持ちなんじゃないかなって思いますよ．				
PtY309	4	仲間意識っていうのもあしますし，ライバルみたいな感じのことも有りますよね．	他患を観察することで自身の励みなる			
PtY310	4	あの人ができたんだったら，私もそんなに変わらないから出来るはずだ，頑張ろうって言う感じで．ネガティブに考えないで，ポジティブに考えると，成果としてもお互い引き上げることが出来ると思います．				
PtY311	5	他の人，全く知らない人とかでも，一生懸命やっているんだなっていうのをみると励みになるというかモチベーションが上がる気がします．	他患の頑張りを観察することで向上する			

PtY312	10	自分と比べるっていうと，1階とかですごく走っている人とかいるじゃないですか.	頑張りを見ることで自身の励みになる	
PtY313	10	なんかあの人達をみるとすごいなって思いますけど，逆になんでまだ退院していないのかなとかって思っちゃいますね.		
PtY314	10	だから目指そうっていう感じではないんですよね.		
PtY315	10	どっちかっていうと，普段一緒に食事をしている方とかでも，まだ大変そうだけど退院が決まって，まだこれからもリハビリするのよとか言っているから，頑張るんだなって，すごいなって思いますね.		
PtY316	10	一人ひとり状態が違うじゃないですか.		
PtY317	10	なので，機能がどうのこうのって言うよりも，その人なりに頑張っているなっていうほうが刺激を受けますよね.		
PtY318	10	みんなそれぞれで自分なりに良くなりたいって思って頑張っているんだなって.		
PtY319	8	周りの人と比べてっていうのももちろんあるよ.	高齢者の頑張りを見ることからの影響	高齢者の努力を見ることの要因
PtY320	8	もう俺よりも年いってて車いすで来たおばちゃんがさ，翌日になって歩いていて，あのおばちゃん歩けるんだってなると，俺もあのおばちゃんより頑張ろうかなってなるじゃん.		

PtY321	8	日常車いすに乗っかっているときに歩いていたおばあちゃんに「大丈夫ですか？」って声をかけられたときは流石にショックだったよ.	
PtY322	8	絶対オレも歩いてやるわって思ったよね.	
PtY323	8	口には出さないけど，あいつよりも良くなろうって思っている人って多いと思うよ.	
PtY324	9	他の人をみて，あの人があれだけ頑張っているんだから俺も頑張ろうっていう気持ちはゼロじゃないですよね.	高齢者の頑張りを見ることからの影響
PtY325	9	自分より年行っている人たちがやっているのを見ると，俺も頑張らないといけないなって感じます.	
PtY326	9	ただ，自分の病気とか状況とかがベースに有るんで，完全に比較は出来ないですけどね.	
PtY327	10	他の患者さんからやる気が変化するっていうことも有りますよ.	高齢患者の訓練様子をみることが励み
PtY328	10	やっぱりその，明らかに若輩者じゃないですか.	
PtY329	10	見ていると年配の方たちもすごく頑張っているなって思うんですよね.	

PtY330	10	回復力とかの面でも若い自分の方が有利ですし．でも皆さん頑張っているなって．		
PtY331	10	頑張っても筋力がつかないんだよねって言いながらも，でも一生懸命にやられているから，その点に関してはやっぱり励まされるっていうのは有りますよね．		
PtY332	8	あいつよりもって思うのは自分より少し上の人を見て思っているんじゃないのかな．	高齢者の能力が高いとやる気が向上する	
PtY333	8	能力とか動きとかが自分よりも出来る人．		
PtY334	8	それよりも年齢が上とかが影響するかな．		
PtY335	8	あのばあちゃん俺よりもすごく歩けてるじゃないかとか思うとすごく思うよね．		
PtY336	6	周りの人が頑張っているから俺も頑張ろうっていう気持ちっていうのはあまりないね．	他患からの影響はない	他患からの影響はない
PtY337	3	競争とかも全くありませんでしたね．	他患と状況が違うため比較は行えない	
PtY338	3	というよりも，自分の競合相手となる人がいなかったというのが正しいですね．		

PtY339	3	競合するということは自分と全く同じ人，症状も年齢も全く同じ．そういう人がいれば競争して，よしやるぞっていうこともあったと思うんです．	相乗効果はあったが，競争はない		
PtY340	3	けど，みんなばらばらですし，そういう人たちと競合してもあまり意味は無いと思うんですよ．			
PtY341	7	周りからの影響っていうよりも自分の目標が有るっていうことが大きいですね．	目標があれば他患からの影響はない		
PtY342	7	同室の人とのやり取りとか，訓練室の人たちとのやり取りとかもそんなに影響しなかったです．			
PtY343	7	やる気が上がることも，下がることもなかったです．			
PtY344	5	他からの影響って言うのは僕自身はわからないっすね．	他患からの影響はない		
PtY345	5	他の人を観察とかはしていないし．周りの人も病気を持っている人なんで．			
PtY346	10	周りからの影響でやる気が無くなるってことはないですね．	周囲からの影響で下がることはない		
PtY347	2	競争というか他の人からの影響っていうのも有ると思います．	患者間交流による相互効果	他患との交流による要因	患者交流による要因

PtY348	2	急性期のときに 6 人部屋だったんですけども，私よりも前から入院していた患者さんがちょうど私と同じぐらいのレベルだったんです．	
PtY349	2	なので，お互いに今日これをやったよとか，これをやったんだよとか話し合いながらっていうのがありました．	
PtY350	2	他の人の訓練で実際に自分でやってみたりとかもしましたね．	
PtY351	2	逆に相手も私がやっている内容をやってみたりして．	
PtY352	2	できない場合はお互いに明日までには出来るようになろうねって言っていました．	
PtY353	2	勝ちたいっていうよりは，お互いに一日も早く退院できるように頑張ろうっていう気持ちがありました．	
PtY354	2	切磋琢磨していたというか．お互いに高め合うことが出来たというか．	
PtY355	2	個室だとそういったことはないですよね．その分自分たちの時間が取れるのはいいことです．	
PtY356	4	個室とかもいいんですけど，大部屋は確かに嫌な部分も有るんですが，それよりも自分の気持を上げてくれるっていう効果があると思います．	同室患者との交流による相乗効果

PtY357	4	自分が上がったんだとしたら，周りの方で落ち込んでいる人がいたとすると，励ましたりもします.	
PtY358	2	他者交流では療法士とか看護師とかとの交流も有ると思いますけど，患者さん同士の交流っていうのは非常に大切だと思います.	患者間交流により維持する
PtY359	2	集団立ち上がり訓練とかでも，今日は何回出来たね，とか挨拶をするのもそうだと思うんですけど，励まし合いとか，お陰様の精神がいいかなって思います.	
PtY360	4	長くいればいるほど，患者さん同士の知り合いも増えますし，その人達が良くなることも大切だと思います.	患者同士の関係の良好さ
PtY361	4	私の周りは幸い良くなっている人が多いので，いい環境で生活できてるなって感じています.	
PtY362	9	患者との関係では，孤立しているよりもいろいろな状況が分かるほうが不安は減ります.	患者交流のでの不安軽減が重要
PtY363	9	今こういう訓練をしているとか，体の状況的にこんな感じなんですとか．今の現状についてシェアできる人がいればいいですよね.	現状の共有が重要
PtY364	9	一人だと不安でしょうがないですよ.	
PtY365	2	療法士の方も近くにいて一番の支えてあることは間違いないのですが，それ以外の患者さんとの交流っていうのも重要かなって思います.	患者同士の交流が心の支え

PtY366	4	高い状態を保てているときっていうのは，一人じゃないって思えた時.	ピアがいることによる安心			
PtY367	4	一人ぼっちじゃないって思ったときですね.				
PtY368	9	自分の病気の場合は家族に助けてもらったんで．助けてもらった命ですからやれるだけ，	家族の存在	家族の存在	支援者の存在の要因	支援者からの要因
PtY369	9	ずっとやり続けないといけないとは思っていますよ.				
PtY370	9	個人の目標っていうよりもそういう周り家族の人達の存在っていうのが圧倒的に大きいです.	周囲の家族の存在により維持			
PtY371	9	個人のためってなるとモチベーションは保たないですよね.				
PtY372	10	娘もいますし，夫と娘の二人の生活もなかなか大変だなって思うので，まあ，なるべく早く帰りたいっていうのがありますよね.	自宅にいる家族のため			
PtY373	9	職場の人とか，家族とかの存在ももちろん大きいですよね.	同僚の存在	同僚の存在		
PtY374	9	会社とか待ってくれている人がいますし，そういう人たちがいるっていうのはモチベーションが高く有り続けられる要因でもありますよね.				

PtY375	6	家族とかにダメ出しされている時とかも落ち込むことも有るけど，そんな馬鹿じゃないぞって思って逆にやる気になったりもしたかな．そこからどうすればいいのかなって考えて，っていう感じかな．	家族の声かけ	家族との会話	支援者との関係性	
PtY376	6	それで成果が出たらまたやる気が上がるしね．やる気の上がるきっかけっていうのは，最初の叱咤があったからかな．				
PtY377	10	疲れたりするとやる気が無くなったりもするんですけど，自分だけだと常にずっとモチベーションが高い状態っていうのは難しいですよね．	他者からの褒めの影響	褒め		
PtY378	10	そういう時って第三者からの励ましが有りましたよね．				
PtY379	10	良くなりましたねとかもそうですし，早く良くなって戻ってきてねとか職場の人からの励ましとかもあるし．そういうのがバランスよくあったんですよね．				
PtY380	10	家族も会社も，もちろん療法士との関係もそうですけどね．	家族や周囲の人達の励ましにより維持			
PtY381	10	でも実際にそれだけではずっと維持するのは難しくて，見舞いに来てくれる人とかの励ましですよね．				
PtY382	10	そういったものがあるとモチベーションが高くなりますよね．				
PtY383	1	とにかくリハビリセンターにいることが多くなります．	高いとき，自主トレをする時間が長くなる	自主トレ時間	自主トレ	行動の変化

PtY384	1	自主トレをするために，ずっといます.	
PtY385	1	毎日いるってことは，毎日モチベーションが高いってことですね.	
PtY386	3	高いときっていうのは，時間は関係なく自分が納得できるまで自主トレとか病棟での訓練をしていますね.	高いとき, 疲れていても自主トレをする
PtY387	3	よくこんなにやってるなって自分が思うぐらいに自発的に時間に関係なく，疲労に関係なくやっていましたね.	
PtY388	3	療法士の先生がついてやってくれる時間は限られていると思うので，今日習ったこととか，うまくできなかったなって思うことなんかを次に先生に見てもらう時には上達しておこうって思うので，自主トレにも熱が入りますよね.	高いとき, 自主トレをして能力向上を図る
PtY389	3	先生から言われたことを自主トレで行って，それを担当の療法士に見てほしいっていう思いが強く有ります.	
PtY390	4	自然と練習するようになりますね.	高いとき, 自主トレを行うことが多くなる
PtY391	4	リハビリの時間だけに頼っていないで, 自分から療法士を待ってみようとか, 廊下での苦手なことをやってみたりとか.	
PtY392	4	リハビリ時間以外の時間も自主トレとか積極的に動くようになっていると思います.	

PtY393	7	高い時っていうのは，自主トレをしましたね.	高い時は自主トレを行う
PtY394	7	昼間にベッドで過ごさずに自分でトレーニングをしていました.	
PtY395	7	時間があると自主トレしていました.	高いときには自主トレを行う
PtY396	7	部屋にいることもあるけども，部屋で出来る自主トレもあるし．寝たきりというか何もしないっていう時間がなかったかな.	
PtY397	9	今は自主トレをしますよね．空いている時間があると自主トレをします.	高いとき，訓練時間以外でも自主トレする
PtY398	9	かみさんと一緒に歩こうかとか，腕に関しても昨日できなかったことを復習してみようとか，自主トレをする回数が圧倒的に増えると思います.	
PtY399	9	訓練中とか以外でも病棟でやりますね.	
PtY400	7	歩き方がおかしいなって思っているときとかは，眠たいから寝ちゃえとか.	低いときは自主トレをしなくなる
PtY401	7	日中時間が有るときにも自主トレをしなかったり，部屋にいても自主トレをしていない時間が増えたり．焦りはあったんだけど，どうしたらいいのかわからないっていうのがありましたね.	

PtY402	9	低いときは，自主トレをしなくなりますね．	低い時は自主トレをしなくなる		
PtY403	9	でも部屋にいることはないです．			
PtY404	9	じっとして部屋にいるほうがいろんなことを考えちゃうんで．悪い方向に考えちゃうんで，そうなるときりがないんで，そうするとリハ室にいったりすると時間もすぎるし自分の能力も上がるし．自分の能力が分かるし，日々の改善も分かりますし．			
PtY405	10	低いときっていうのは高いときの逆で，自主トレは面倒くさいなって思ったり，疲れているからいいかなとか．	低い時は自主トレはしなくなる		
PtY406	8	時間だけはたっぷりあるからな．これをベッドに横になってテレビを見ているだけっていうのもつまらないしさ．だったら動くかってなるじゃん．	高いときは部屋にいる時間が減る	自室滞在時間	能動的動作の増加
PtY407	8	俺自身客商売やっていたから人を見るのよ．人をみて自主トレ方法とか，療法士が言っていることを小耳に挟むとだんだん自分もやらないとなとか自分もそうなるんだなとか思うじゃん．			
PtY408	8	やっぱりさ，ベッドにいちゃダメなんだよ．この病気になったら時間だけはあるんだからさ，自分で動かないとダメだよ．とりあえず出てって，人がやっていることを見ているってのは大切だと思うんだよ．			
PtY409	8	あの人あんなに苦しそうだけど，あんなに頑張っているんだとか思うじゃん．それで自分もやろうかなって思うわけ．			
PtY410	10	足繁く売店に行ってみたりとかもあるだろうし，うろうろしてみたり，他の人を見てみたり．	高いときには自室以外にいることが多い		

PtY411	1	やる気が無いときっていうのはリハビリセンターに来ることがなくなるというか，回数は減るでしょうね．	低いときは部屋にいる時間が増える	
PtY412	1	自分の部屋にいる時間が増えるというか．部屋にいて電話とかしている時間が増えると思いますね．		
PtY413	1	あまり外に出ないし，悪循環にハマっているような感覚になると思いますね．		
PtY414	6	あとは部屋で悩んでいる時間が増えていたっていうのもあるかな．	低い時は部屋で悩む時間が増える	
PtY415	6	このままじゃ家に帰れないなって．		
PtY416	1	いろいろな人と話はするんですけど，ぽつんと一人でいるとか，部屋にこもっている人とかは悲観的になっちゃっているなって思います．	部屋にいる時は悲観的な環状になる	
PtY417	6	モチベーションが上がっているときっていうのは，積極的に療法士に昨日行った道をもう一度行ってくれって頼んだりしていたかな．	高いときは能動的に訓練に参加する	訓練への能動的態度
PtY418	6	自分から積極的に頼んでいたかな．自分の要望を伝えてたかな．		
PtY419	10	訓練のときとかも退院が近いのでこれから何をしたら良いですかとか，自主トレどうしたらいいですかとか聞きますよね．	高いときには自主トレ方法などを聞く	

PtY420	6	低いときっていうのは，何もやる気しなかったな.	低いときには何もやる気にならない
PtY421	6	何やっても面白くないし.	
PtY422	4	そうじゃない時には，呼ばれるまで待っていて呼ばれたから行って，そして帰ってきたら寝てとかいう感じだったんですけど，寝ないで座っていようとか．自分の姿勢が変わってきていたと思います.	低いときは訓練に消極的であり受動的
PtY423	4	低いときっていうのは，引きずられているような感覚ですかね.	
PtY424	4	先生が一生懸命に引っ張っていてくれているのにそれに引きずられている感じ.	
PtY425	4	呼ばれたから行くとか，言われたからやるとか.	
PtY426	6	あまりよくないなって感じたけど．療法士がついている訓練のときにもあまり積極的じゃないし.	低いときは難易度が低い内容を好む
PtY427	6	出来そうだなって思うことしかやらなくなっていたっていうのも有るね.	
PtY428	6	失敗したくないっていう気持ちもあったし．頭の練習じゃなくて，手の麻痺を治すような運動のリハビリばっかりをやっていたな.	

PtY429	6	それだと道を覚えるとかのリハビリと違って失敗しないし.			
PtY430	2	モチベーションが高いときって言うのはいつも笑顔でいられる.	高いときは表情が良い	表情	表情，言動
PtY431	2	笑顔が多いときっていうのは何か自分の中でできなかったことが出来るようになったとか，そういうことが合ったと思うんです.	高いときには笑顔が多くなる		
PtY432	2	あとは，眼力．顔とか，表情とかから相手の心理状態がわかると思うんです.			
PtY433	6	あとは笑顔がなくなったかな．表情に出ていたと思う.	低いときは笑顔が減る		
PtY434	2	行動としても，歩き方も元気な歩き方というか.	低いときは動作の質が下がっている	動作の表出	
PtY435	2	ダメなときは，ダメなのダメなのっていう歩き方になっていると思います.			
PtY436	2	歩き方が早いとか遅いっていうのはその人々で違うんですけど，元気な歩き方というかそういう雰囲気.			
PtY437	3	やる気が低いときには，体が動かないことが多かったかな.	低いときには動作の質が低下する		

PtY438	3	私の場合は痛みがあるときが多いので，臆病になってしまうんですよね.	やる気無いときは動作遂行能力が下がる	
PtY439	3	例えば，立ち上がったときとかに痛いとかが続いてしまうと，体が拒否をするんですよ.		
PtY440	3	本来は動ける動作でも，もしかしたら痛くなるかもって考えるとすごく慎重になってしまいます.		
PtY441	3	動きは遅くなるし，杖を使わなくてもいいのに，杖を使っちゃうし.	低いときには動作能力が低下する	
PtY442	3	悪いとことと言うか，痛みがあると，体が動かなくなってしまって，それまで自分が思っていたよりも多く訓練をしていたのに，少なく安全に行うようになってしまいます.		
PtY443	3	運動量は確実に減りました.		
PtY444	1	ずっと来ていると常連の患者さんとも仲良くなるじゃないですか，そうするとそこで情報交換とかしたりしますね.	高いとき，患者同士の交流を積極的に持つ	他患との会話
PtY445	1	やる気があるときっていうのは，同じような境遇の人と交流を持とうとします.	高いとき，状況が類似する人と交流を持つ	
PtY446	2	後はモチベーションが高いときっていうのは人とおしゃべりがしたくなります.	高いときには他者交流を積極的に行う	

PtY447	2	他者との交流を持ちたくなることが多いですし，おばあちゃんとかで話しかけてくれる人をみるとモチベーションが高いのかなって感じます.	
PtY448	2	お食事の仲間とかでも話しかけられたくないっていう人もいたりします.	
PtY449	2	でも，一期一会ですし，その時間を使って手助けしてあげたり，したいなって思いますし，私自身が助けられうこともあります	
PtY450	1	私自身は人と話すことが好きなので，看護師さんとか患者さんと話をすることで気分が高まることも多いです.	高いとき, 他者と交流を持つことが多い
PtY451	10	一緒に御飯を食べている人達に自分から話しかけることもあったし，家族や友人に情報発信したり，メールとかラインとかしてみたり．積極的に発信するっていう感じですよね.	高いときには情報発信を積極的に行う
PtY452	10	高い時って言うのは自主トレもやってみたりするし，自分からいろんな人達にアクションをすることも有りましたね.	高いとき, 他者との交流を持つことが多い
PtY453	10	療法士とか看護師とかに対してもあるかもしれないですよね.	
PtY454	10	普段より多く話しかけてみたりっていうこととか.	
PtY455	2	モチベーションが低い時っていうのは割りと口数が少なくなったりすると思います.	低いときには他者交流が減少する

PtY456	2	元気がない時は話すことすらしたくない.		
PtY457	10	引きこもるってことはなかったですけど，でもここにいると時間になればリハビリが入ってきますからね.	低いときにはアウトプットが少なくなる	
PtY458	10	自分からの発信がなくなっていくとか，アウトプットしなくなるとか.		
PtY459	10	でもやる気が無ければあえて自分から部屋から出ようっていう気持ちにはならないんじゃないんですかね.		
PtY460	5	高いときも低いときも特別行動が変わるってことはないですね.	高いときも低いときも変化は少ない	行動変化なし
PtY461	5	あまり，目に見えて行動が変わるってことはないです.		
PtY462	5	表情とか言動とかに関しても大きくは変わってないと思います.		
